# Mitochondria‐specific nanocatalysts for chemotherapy‐augmented sequential chemoreactive tumor therapy

**DOI:** 10.1002/EXP.20210149

**Published:** 2021-09-01

**Authors:** Hui Huang, Caihong Dong, Meiqi Chang, Li Ding, Liang Chen, Wei Feng, Yu Chen

**Affiliations:** ^1^ School of Environmental and Chemical Engineering Shanghai University Shanghai P. R. China; ^2^ Materdicine Lab, School of Life Sciences Shanghai University Shanghai P. R. China; ^3^ State Key Laboratory of High Performance Ceramics and Superfine Microstructure, Shanghai Institute of Ceramics Chinese Academy of Sciences Shanghai P. R. China; ^4^ Department of Ultrasound, Zhongshan Hospital Fudan University, and Shanghai Institute of Medical Imaging Shanghai P. R. China

**Keywords:** autophagy, chemodynamic therapy, cisplatin prodrug, mitochondria‐specific nanocatalysts

## Abstract

Endogenic tumor chemodynamic therapy (CDT) is emerging as a tumor‐therapeutic strategy featuring in situ treatments with high efficiency and specificity based on the Fenton reaction principle. Considering the limitation of monotherapy and relatively insufficient intracellular level of endogenous hydrogen peroxide (H_2_O_2_) in tumor tissues, a mitochondria‐specific nanocatalyst composed of cisplatin prodrug and gallic acid‐ferrous (GA‐Fe(II)) nanocomposites is successfully fabricated to fulfill chemotherapy‐augmented sequential chemoreactive tumor therapy. The bioactive cisplatin elevates the level of endogenous H_2_O_2_ through the activation of nicotinamide adenine dinucleotide phosphate oxidase (NOX)‐related cascaded reactions, and the GA‐Fe(II) nanocomposites possessing sustainable Fenton catalytic activity subsequently catalyzes H_2_O_2_ into highly reactive and toxic hydroxyl radicals to substantially inhibit tumor progression. Especially, this mitochondria‐specific nanocatalyst amplifies oxidative stress, stimulates mitochondrial dysfunction, downregulates AKT/mTOR signaling and finally induces cell autophagic death. Both in vitro and in vivo measurements verify that the chemotherapy‐augmented sequential chemoreactive nanotherapy based on the mitochondria‐specific nanocatalyst implements excellent anticancer efficiency and avoids undesired side effects. This work reveals the enormous potential of chemotherapy‐augmented CDT for combating tumors.

## Graphical abstract

The therapeutic modality of “chemotherapy‐augmented sequential chemoreactive therapy” based on the designed mitochondria‐specific nanocatalyst composed of cisplatin prodrug and gallic acid‐ferrous (GA‐Fe(II)) nanocomposites achieves distinct catalytic cancer‐therapeutic performance. The bioactive cisplatin elevates the endogenous H_2_O_2_ level through the cascaded reactions, and the GA‐Fe(II) nanocomposites with sustainable Fenton catalytic activity subsequently catalyzes H_2_O_2_ into hydroxyl radicals to substantially inhibit tumor progression.

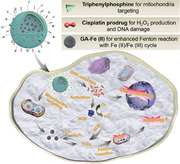

## 1 INTRODUCTION

Nanodynamic therapy typically refers to the integration of nanomedicine with varied dynamic therapies to implement different therapeutic purposes, such as chemodynamic therapy (CDT), photodynamic therapy, sonodynamic therapy, and so on.^[^ ^1^ ^]^ Among them, CDT, as an emerging therapeutic strategy, typically takes the advantage of Fe(II) ions‐mediated Fenton reaction for converting endogenous hydrogen peroxide (H_2_O_2_) into highly reactive and toxic hydroxyl radicals (•OH) with the strongest oxidative capability (*E*(•OH/H_2_O) = 2.80 V) among the common reactive oxygen species (ROS) inclusive of singlet oxygen (^1^O_2_, *E*(^1^O_2_/H_2_O) = 2.17 V) and H_2_O_2_ (*E*(H_2_O_2_/H_2_O) = 1.78 V), which represents as a safe and effective tumor‐therapeutic modality.^[^ ^2^ ^]^ Obviously, the generation of •OH through the typical Fenton catalysis process predominantly depends on the reaction between the catalysts and endogenous H_2_O_2_ under the favorable acidic condition without requiring external energy and oxygen (O_2_). Despite the intracellularly generated H_2_O_2_ levels in tumor cells were higher than that in normal cells due to the altered metabolism, the endogenous H_2_O_2_ level is still insufficient to achieve satisfactory CDT efficacy.^[^ ^3^ ^]^ In this aspect, it is highly desirable to improve the endogenous H_2_O_2_ supply in the tumor microenvironment (TME) for elevating CDT antitumor efficiency based on the Fenton reaction.

Apart from exogenous H_2_O_2_ delivery in TME, the amplification of endogenous H_2_O_2_ generation in mitochondria is regarded as another important strategy to elevate the intracellular H_2_O_2_ levels. Platinum compounds, such as cisplatin, play a significant role in tumor chemotherapy as utilized in ∼80% of clinical regimens, where the chemotherapeutic effects are strongly related to the formation of ROS.^[^ ^4^ ^]^ Specifically, cisplatin can result in successive activation reactions involving enzymes, namely nicotinamide adenine dinucleotide phosphate (NADPH) oxidase (NOX), which subsequently facilitates the conversion from NADPH into NADP accompanied with the electrons release.^[^ ^5^ ^]^ In turn, the O_2_ molecule accepts a donated electron to generate O_2_^•−^, and is subsequently dismutated by superoxide dismutase enzyme (SOD) to produce H_2_O_2_, thus providing sufficient reactants to meet the requirement of the Fenton reaction.^[^ ^5^, ^6^ ^]^ Nevertheless, platinum‐based chemotherapy inevitably leads to severe side effects, such as nephrotoxicity and ototoxicity.^[^ ^7^ ^]^ Constructing TME‐responsive and mitochondrial‐targeting drug delivery system not only improves the biosafety of platinum drugs to reduce the side effects, but also facilitates the site‐specific conversion from O_2_ to H_2_O_2_ to maximize antitumor efficacy. Moreover, in Fe(II)‐mediated Fenton catalysis process, H_2_O_2_ is catalyzed by Fe(II) to form •OH, and concurrently Fe(II) is oxidized into Fe(III).^[^ ^8^ ^]^ It is demonstrated that Fe(III) ions possess lower catalytic activity than Fe(II) ions, and require high activation energy to form Fe(II), which is the substantial rate‐determining step for the Fenton reaction.^[^ ^9^ ^]^ Considerable efforts have been devoted to accelerating the regeneration rate of Fe(II) inclusive of integrating reductive substances or semiconductor materials with Fe‐containing catalysts for augmented Fenton reaction.^[^ ^2^ ^e,^ ^10^ ^]^ However, the strategy that electron transfers from light‐excited semiconductor materials to Fe(III) is hindered by the poor tissue penetration depth of light radiation.^[^ ^8^ ^]^ Beyond that, integrating reductive substances into the Fenton catalytic system holds high promise for enhancing the therapeutic performance of CDT.

**FIGURE 1:**
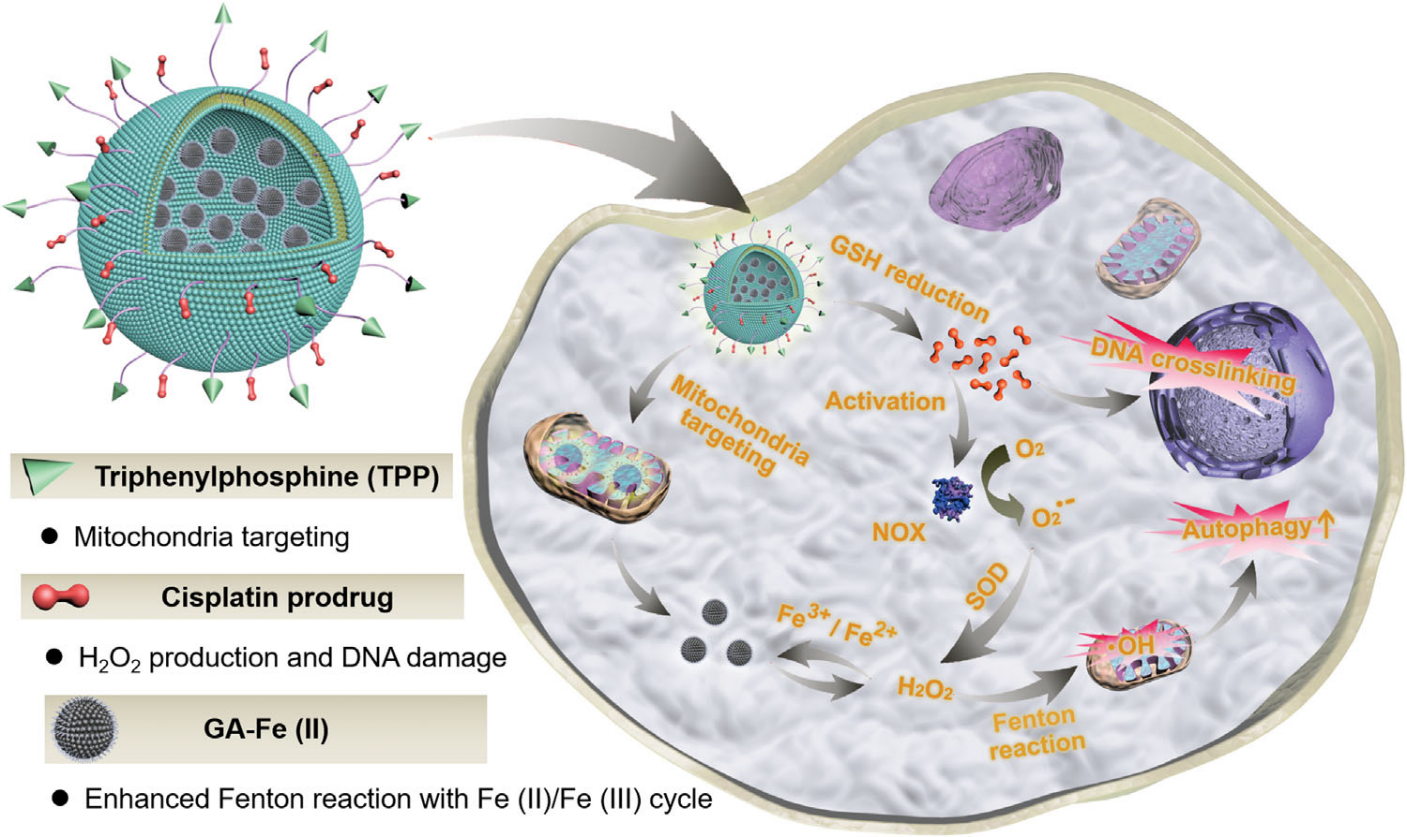
Schematic diagram of the in vivo chemotherapy‐augmented sequential chemodynamic tumor therapy based on mitochondria‐specific nanocatalysts. The bioactive cisplatin elevates the level of endogenous H_2_O_2_ through the activation of NOX‐associated cascaded reactions, and the integrated GA‐Fe(II) nanocomposites with sustainable and efficient catalytic activity subsequently catalyze H_2_O_2_ into highly toxic hydroxyl radical to induce autophagy for substantially inhibiting tumor progression

Herein, we report on a proof‐of‐concept “chemotherapy‐enhanced sequential chemoreactive therapy” for combating cancer, which dramatically elevates the catalytic efficiency of Fenton reaction for efficient •OH production and accordingly inhibits tumor growth. In this study, a biocompatible and stable Fenton catalyst was initially fabricated via mixing Fe(II) ions with gallic acid (GA) to obtain GA‐Fe(II) nanocomposites. Then, GA‐Fe(II) and cisplatin(IV) prodrugs were successfully co‐encapsulated within mitochondria‐targeting liposomes to construct multifunctional composite nanocatalysts (designed as Pt/GF@Lipo‐TPP). The design of Pt/GF@Lipo‐TPP is based on the following considerations (Figure 1). (i) Cisplatin can activate NOX to promote the conversion from O_2_ to H_2_O_2_, providing sufficient H_2_O_2_ for the subsequent Fenton reaction and fulfilling chemotherapy‐enhanced sequential CDT‐based chemoreactive therapy. Furthermore, the glutathione (GSH)‐responsive drug delivery/release can lower the cisplatin‐induced concentration‐dependent toxicity in practices. (ii) The synthesized GA‐Fe(II) nanocomposites exhibit sustainable and efficient catalytic effect in converting H_2_O_2_ to toxic •OH and significantly enhance the catalytic stability of Fe(II) ions ascribing to the GA‐mediated Fe(III) to Fe(II) conversion. (iii) Mitochondria‐specific drug delivery and targeting strategies of •OH formation have been utilized to amplify the cellular oxidative stress and lead to tumor cell death, which desirably boosts the tumor therapeutic efficiency of chemotherapy‐augmented sequential CDT.

## 2 RESULTS AND DISCUSSION

### 2.1 Materials synthesis and characterization

**FIGURE 2:**
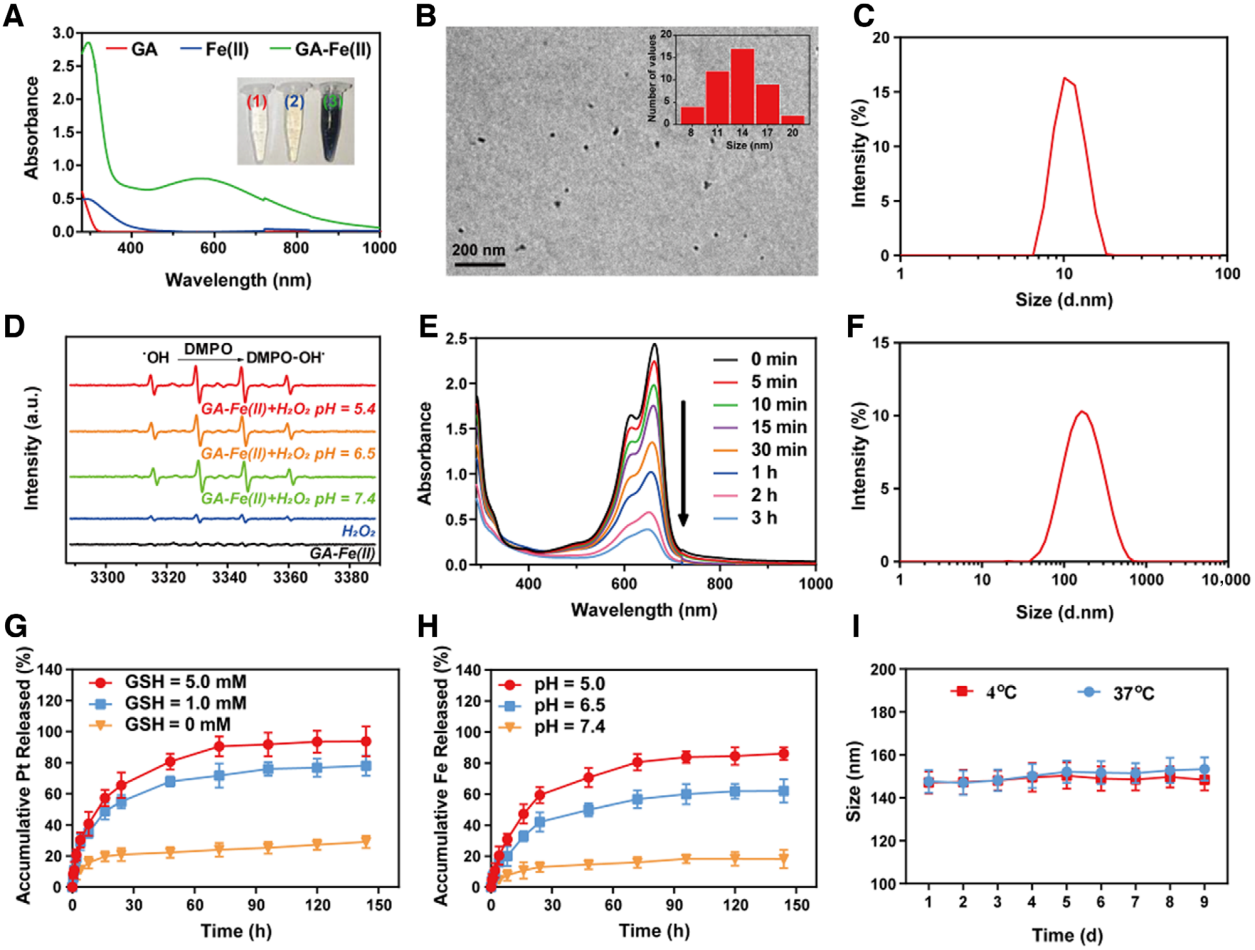
Fabrication and characterization of Pt/GF@Lipo‐TPP. (A) Schematic illustration of synthetic process regarding Pt/GF@Lipo‐TPP. (B) TEM imaging of GA‐Fe(II) nanocomposites and corresponding size distribution (inset). (C) DLS analysis of GA‐Fe(II) nanocomposites. (D) ESR spectra of GA‐Fe(II) at different pH values. (E) The UV–vis–NIR spectrum of MB degradation induced by GA‐Fe(II) nanocomposites in a time‐dependent manner. (F) Size distribution of Pt/GF@Lipo‐TPP by DLS analysis. (G) The release profile of Pt from Pt/GF@Lipo‐TPP in the absence and presence of GSH. (H) The release profile of Fe from Pt/GF@Lipo‐TPP at different pH values. (I) Size change of Pt/GF@Lipo‐TPP at 4 and 37°C

We initially successfully synthesized 1,2‐distearoyl‐*sn*‐glycero‐3‐phosphoethanolamine‐*N*‐(polyethylene glycol)_2000_‐Pt(IV) (DSPE‐PEG_2k_‐Pt(IV)) and 1,2‐distearoyl‐*sn*‐glycero‐3‐phosphoethanolamine‐*N*‐(polyethylene glycol)_5000_‐triphenylphosphonium bromide (DSPE‐PEG_5k_‐TPP), which were confirmed by the results of nuclear magnetic resonance (^1^H NMR) (Figures S1–S4). Then, the ultrasmall GA‐Fe(II) nanocomposites were constructed through the strong coordination interactions between Fe(II) ions and the polyphenol groups of GA. After adding GA aqueous solution into the mixed solution of iron(II) dichloride (FeCl_2_) and polyvinylpyrrolidone (PVP), the color of the mixed solution immediately transferred from colorless to purple (Figure 2A). The Fourier transform infrared spectrum demonstrates a decrease in the band of GA‐Fe(II) at 1250 cm^−1^ associated with the OH─C stretching band, confirming the coordination interaction between Fe(II) ions and ─OH groups of GA (Figure S5). The dynamic light scattering (DLS) analysis and transmission electron microscopy (TEM) image indicate that the GA‐Fe(II) nanocomposites are gifted with discrete distribution and uniform size possessing an average diameter at ∼13 nm (Figure 2B,C). The zeta potential analysis demonstrates that the surface potential of Pt/GF@Lipo‐TPP is −24.4 ± 0.21 mV in an aqueous solution. Furthermore, in order to investigate the catalytic activity of GA‐Fe(II) nanocomposites as Fenton catalysts, the •OH formation was measured through the electron spin resonance (ESR) spectroscopy assisted with 5,5‐dimethyl‐1‐pyrroline‐*N*‐oxide (DMPO), detection of the short‐lived •OH species.^[^ ^11^ ^]^ In the ESR spectrum, the characteristic 1:2:2:1 signals are only emerged in the GA‐Fe(II) nanocomposites incubated with H_2_O_2_, suggesting the efficient •OH production (Figure 2D). Moreover, the ESR signals enhance with decreasing pH value, indicating the pH‐sensitive catalytic property of GA‐Fe(II) nanocomposites. In addition to ESR, methylene blue (MB) that is generally applied as an indicator was utilized to measure the formation of •OH. The color of MB solution in the presence of GA‐Fe(II) nanocomposites and H_2_O_2_ gradually changes from blue to colorlessness in a time‐related degradation manner, further demonstrating the formation of •OH (Figure 2E).

Subsequently, the as‐fabricated GA‐Fe(II) nanocomposites were encapsulated within stealthy liposomes (named Pt/GF@Lipo‐TPP) through hydrating the formed lipid film consisting of cholesterol, 1,2‐dihexadecanoyl‐sn‐glycero‐3‐phosphocholine (DPPC), DSPE‐PEG_2k_‐Pt(IV) and DSPE‐PEG_5k_‐TPP. As demonstrated under DLS measurement and TEM imaging, the obtained Pt/GF@Lipo‐TPP exhibits spherical morphology and monodispersed size distribution with the average diameter at ∼100 nm (Figure 2F; Figure S6). The release profile of bioactive Pt and Fe elements were studied under different glutathione (GSH) and pH levels via a dialysis method (Figure 2G,H). In the absence of GSH, only ∼20% Pt and Fe are released within 72 h, demonstrating that the Pt/GF@Lipo‐TPP is stable at pH 7.4 (Figure 2G), whereas with the aid of GSH, the release rate of the Pt element is substantially elevated. After 72 h incubation, the cumulative release percentages of Pt from Pt/GF@Lipo‐TPP at 1.0 or 5.0 mm GSH were determined as 71.67 ± 7.69% and 90.50 ± 6.36%, respectively, suggesting GSH‐responsive prodrug release. Because of the disassembly of GA‐Fe(II) nanocomposites in virtue of the protonation of polyphenols, burst release of Fe is detected under the acidic condition (Figure 2H). In blood circulation, the physiological characteristics including low GSH concentration (1–10 μm) and relatively high pH value enable Pt/GF@Lipo‐TPP with stability and inactivation, which are tailored for long circulation. At tumor tissue, the intracellular GSH concentration is much higher than that at normal tissue,^[^ ^12^ ^]^ therefore in response to the signals of elevated GSH level and relatively low pH in TME, the release rate of Pt and Fe elements can be dramatically accelerated, implementing site‐specific drug delivery and avoiding undesired side effects. The average size of the as‐prepared Pt/GF@Lipo‐TPP shows ignorable change within 9 days at 4, 25, or even 37°C, indicating excellent storage stability (Figure 2I; Figure S7).

### 2.2 Intracellular fate of Pt/GF@Lipo‐TPP

**FIGURE 3:**
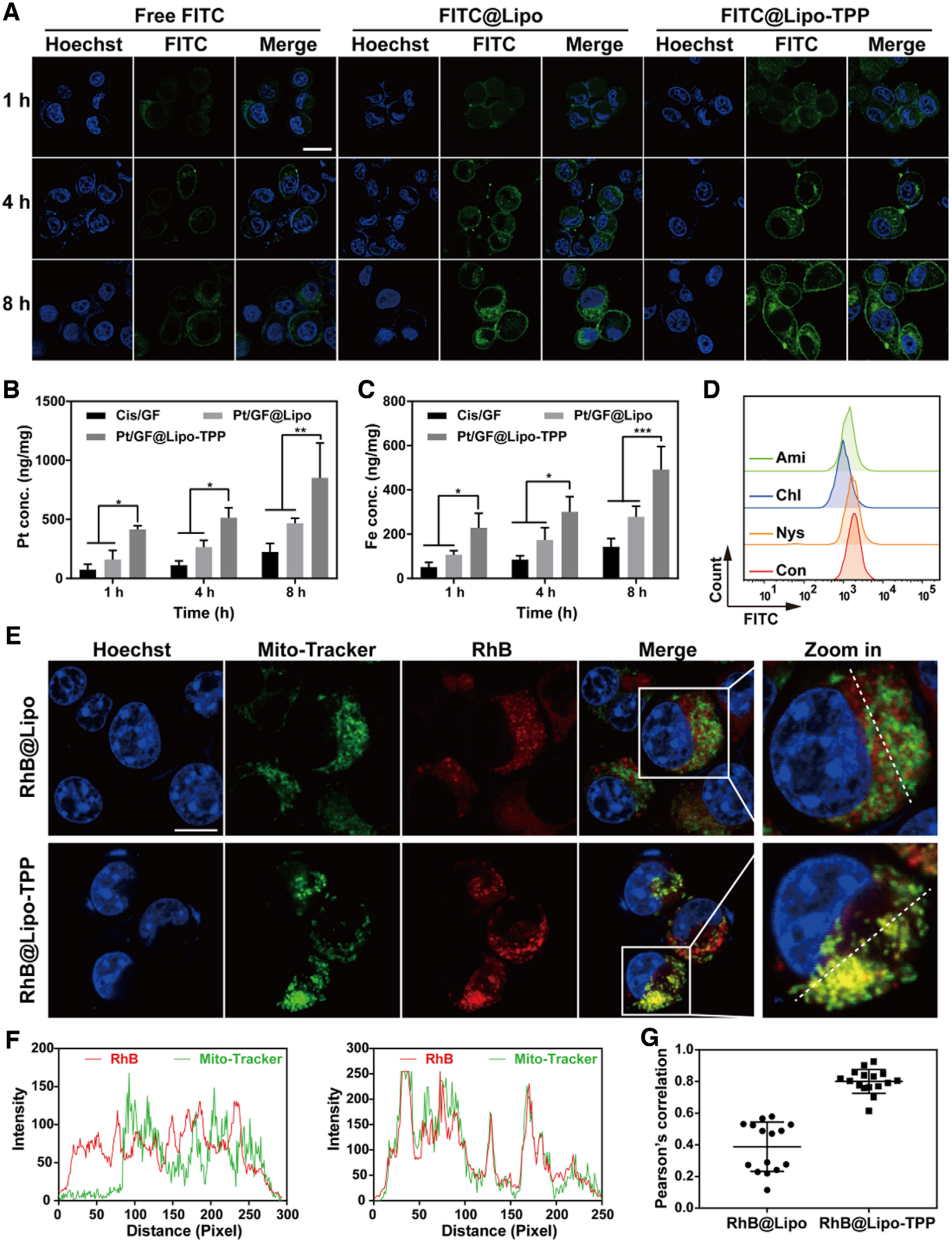
The intracellular fate of Pt/GF@Lipo‐TPP in 4T1 cells. (A) The cellular uptake measured by CLSM images of 4T1 tumor cells treated with free FITC, FITC@Lipo, and FITC@Lipo‐TPP for 1, 4, and 8 h, respectively. Scale bars = 20 μm. (B and C) The intracellular concentration of Pt (B) and Fe (C) after cells incubated with different formulations for different durations. (D) Flow cytometer analysis of 4T1 cells pretreated with different endocytic inhibitors including Nys, Chl, and Ami. (E) Colocalization of mitochondria with RhB@Lipo or RhB@Lipo‐TPP by CLSM images. (F) The fluorescent intensity along the white dotted line in (E) analyzed by the Image J software. (G) The colocalization coefficient of mitochondria with RhB@Lipo or RhB@Lipo‐TPP on the basis of CLSM images

Because the intracellular fate of Pt/GF@Lipo‐TPP is crucial for their organelle‐specific delivery, the liposomes were labeled with fluorescein isothiocyanate (FITC) or Rhodamine B (RhB) dye and observed on a confocal laser scanning microscope (CLSM) for visualizing their cellular behavior in 4T1 cells (Figure 3A). The FITC fluorescent signals are weak in tumor cells after co‐cultured for 1 h, suggesting the limited cellular internalization of all formulations in a short time period. After incubation for 4 h, the fluorescent intensity is obviously enhanced and the fluorescent intensity of FITC@Lipo‐TPP treated with 4T1 cells is much stronger than that of FITC and FITC@Lipo groups, respectively. Notably, most FITC@Lipo‐TPP is distributed in the cytoplasm after 8 h, indicating the efficient cellular internalization of Pt/GF@Lipo‐TPP. The results of the flow cytometer analysis are consistent with the CLSM investigation (Figure S8). In addition, the endocytosis of Pt/GF@Lipo‐TPP was further verified using inductively coupled plasma optical emission spectrometry (ICP‐OES). As illustrated in Figure 3B,C, the uptake of Pt/GF@Lipo‐TPP by 4T1 cells displays a time‐dependent manner. The intracellular concentration of Pt and Fe after incubation with Pt/GF@Lipo‐TPP is much higher than cisplatin/GA‐Fe(II) mixture (Cis/GF) and Pt/GF@Lipo.

Subsequently, the endocytic mechanisms of Pt/GF@Lipo‐TPP were studied using CLSM through pretreating 4T1 cells with varied endocytic inhibitors including chlorpromazine (Chl), amiloride (Ami), and nystatin (Nys). Compared with the apparent green fluorescence as observed in the cytoplasm after pretreatment with Nys for 4 h, the fluorescent signals of 4T1 cells pretreated with Chl and Ami almost disappeared (Figure S9). Additionally, the flow cytometer analysis was further applied to quantitatively confirm the cellular internalization pathways of Pt/GF@Lipo‐TPP (Figure 3D). Pretreatment of 4T1 cells with Nys, Chl, or Ami results in an 8.2%, 42.7%, or 27.3% reduction of the cellular internalization, respectively, manifesting that Pt/GF@Lipo‐TPP is internalized predominantly through clathrin‐mediated endocytosis and macropinocytosis.^[^ ^12^, ^13^ ^]^ Finally, we further verified the mitochondria‐targeting efficiency of Pt/GF@Lipo‐TPP. Following the mitochondria colocalization of RhB@Lipo and RhB@Lipo‐TPP on 4T1 cells for 4 h (Figure 3E), the mitochondria with the punctate or rod‐like structure are labeled with green fluorescence, and the strong yellow fluorescent signals (merged by red and green fluorescent signals) are evidently observed in 4T1 cancer cells after incubation with RhB@Lipo‐TPP (Figure 3E,F). Moreover, the colocalization coefficient of RhB@Lipo‐TPP with mitochondria is remarkably higher than that of RhB@Lipo (Figure 3G). Based on the aforementioned results, it is confirmed that Pt/GF@Lipo‐TPP could achieve effective mitochondria targeting in tumor cells, attributing to the functionalization of lipophilic cation TPP.^[^ ^14^ ^]^

### 2.3 Amplification of oxidative stress by Pt/GF@Lipo‐TPP in mitochondria

**FIGURE 4:**
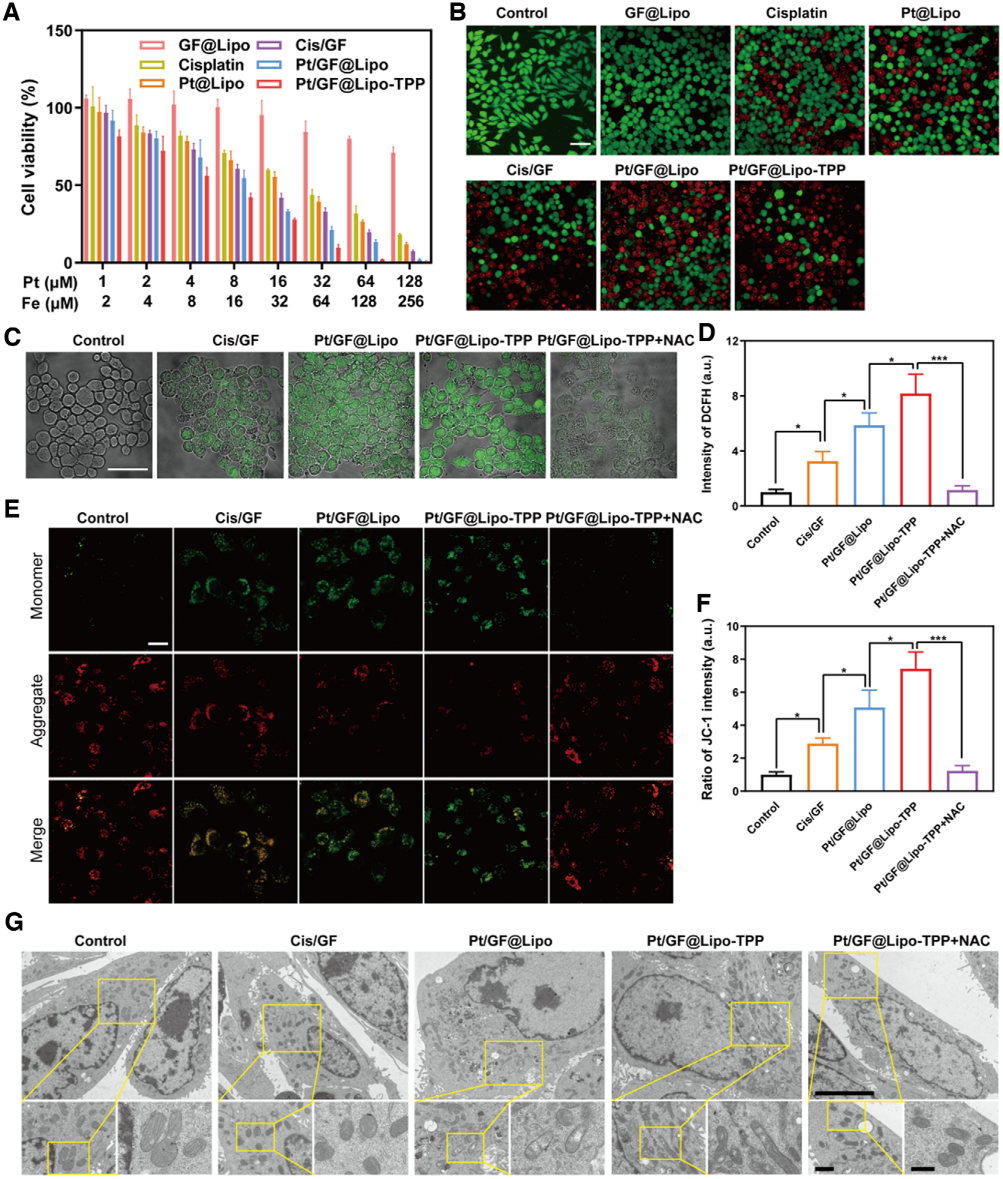
Amplification of oxidative stress by Pt/GF@Lipo‐TPP in mitochondria. (A) Cell viability of 4T1 cells incubated with various treatments for 24 h. (B) CLSM images of 4T1 tumor cells stained with calcein‐AM/PI for 24 h. Scale bars = 50 μm. (C and D) The ROS level utilizing DCFH‐DA fluorescent dye after different treatments detected by CLSM (C) and flow cytometry (D). Scale bars = 50 μm. (E and F) Mitochondrial membrane potential using JC‐1 fluorescent probe by CLSM (E) and the corresponding green (monomer)/red (aggregate) fluorescent intensity ratio (F). (G) Mitochondria ultrastructure in 4T1 tumor cells after treatment with different formulations by Bio‐TEM at low (scale bars = 5 μm), middle (scale bars = 1 μm), and high (scale bars = 500 nm) magnifications, respectively

The in vitro cytotoxicity of different formulations on 4T1 cells was next investigated using the standard cell counting kit‐8 (CCK‐8) assay (Figure 4A; Figure S10). As shown in Figure 4A, these formulations display both time‐ and concentration‐dependent antitumor activity at 24 h, in which Pt/GF@Lipo‐TPP exhibits the highest level of synergistic therapeutic efficiency, indicating the significantly augmented CDT therapeutic performance by chemotherapy based on mitochondria‐specific nanocatalysts. The chemotherapy‐augmented sequential CDT performance was further examined by co‐staining calcine AM and propidium iodide (PI) for living and dead cells, respectively (Figure 4B; Figure S11). In comparison with the control group, a significant reduction in viability is observed in 4T1 cells incubated with Pt/GF@Lipo‐TPP, which is in accordance with the CCK‐8 results. Similarly, the intracellular Pt‐DNA adducts substantially elevated in 4T1 cells after treatment with Pt/GF@Lipo‐TPP (Figure S12). As mentioned above, the released bioactive cisplatin triggers the activation of NOX to transfer O_2_ into H_2_O_2_ via cascade reactions, and H_2_O_2_ is subsequently catalyzed into toxic •OH on the basis of the Fe^2+^‐mediated Fenton reaction to amplify the intracellular oxidation stress. Thereupon, the intracellular formation of ROS was measured by using a fluorescent probe 2,7‐dichlorofluorescin diacetate (Figure 4C). The green fluorescent intensity of the cells incubated with Pt/GF@Lipo‐TPP exhibits a significant enhancement compared with other groups, demonstrating the substantially elevated ROS level. Furthermore, the results of the flow cytometer analysis verify 3.26‐, 5.86‐, and 8.18‐fold increment of ROS in the 4T1 cells cultured with Cis/GF, Pt/GF@Lipo, and Pt/GF@Lipo‐TPP, respectively, confirming the amplification of ROS production by integrating cisplatin and GA‐Fe(II) nanocomposites (Figure 4D). Pt/GF@Lipo‐TPP produced more ROS than the mixture of cisplatin and GA‐Fe(II), owing to the following reasons: (i) Pt/GF@Lipo‐TPP can be efficiently internalized by tumor cells via endocytosis; (ii) Mitochondria‐targeting delivery significantly amplifies the oxidative stress caused by •OH; (iii) The cellular internalization rate and amount of cisplatin and GA‐Fe nanocomposites are different, which impedes the production of ROS.

It is well known that high levels of ROS can induce the oxidation stress of biological molecules containing the reduction of mitochondrial membrane potential, which results in irreversible damage to mitochondria and further inducing cell death. To elucidate the anticancer mechanism of Pt/GF@Lipo‐TPP, 5,5′,6,6′‐tetrachloro‐1,1′,3,3′‐tetraethylbenzimidazolylcarbocyanine iodide (JC‐1 dyes), as an indicator of mitochondrial depolarization evidenced by elevation in the green (monomer)/red (aggregate) fluorescent intensity ratio, was applied to detect the mitochondrial membrane potential. The fluorescent intensity ratio of green to red considerably increases in 4T1 cells after incubation with Pt/GF@Lipo‐TPP, validating the dramatic reduction of the mitochondrial membrane potential (Figure 4E,F). Subsequently, we observed the mitochondrial ultrastructure through bio‐TEM (Figure 4G), showing that the mitochondria in the control group are characterized with the clear and intact double‐membrane structure without apparent swelling, whereas the mitochondria in 4T1 cells incubated with Pt/GF@Lipo and Pt/GF@Lipo‐TPP exhibits noticeable swell, cristae fragmentation and degeneration, particularly in Pt/GF@Lipo‐TPP group, which discloses that Pt/GF@Lipo‐TPP treatment induced mitochondrial damage and disrupted mitochondrial morphology. Especially, the mitochondrial membrane potential changes and mitochondrial damage induced by Pt/GF@Lipo‐TPP can be evidently recovered by *N*‐Acetyl‐l‐cysteine (NAC, a ROS scavenger), confirming the important role of excessive oxidative stress in promoting mitochondria dysfunction.

### 2.4 Autophagy induced by Pt/GF@Lipo‐TPP

**FIGURE 5:**
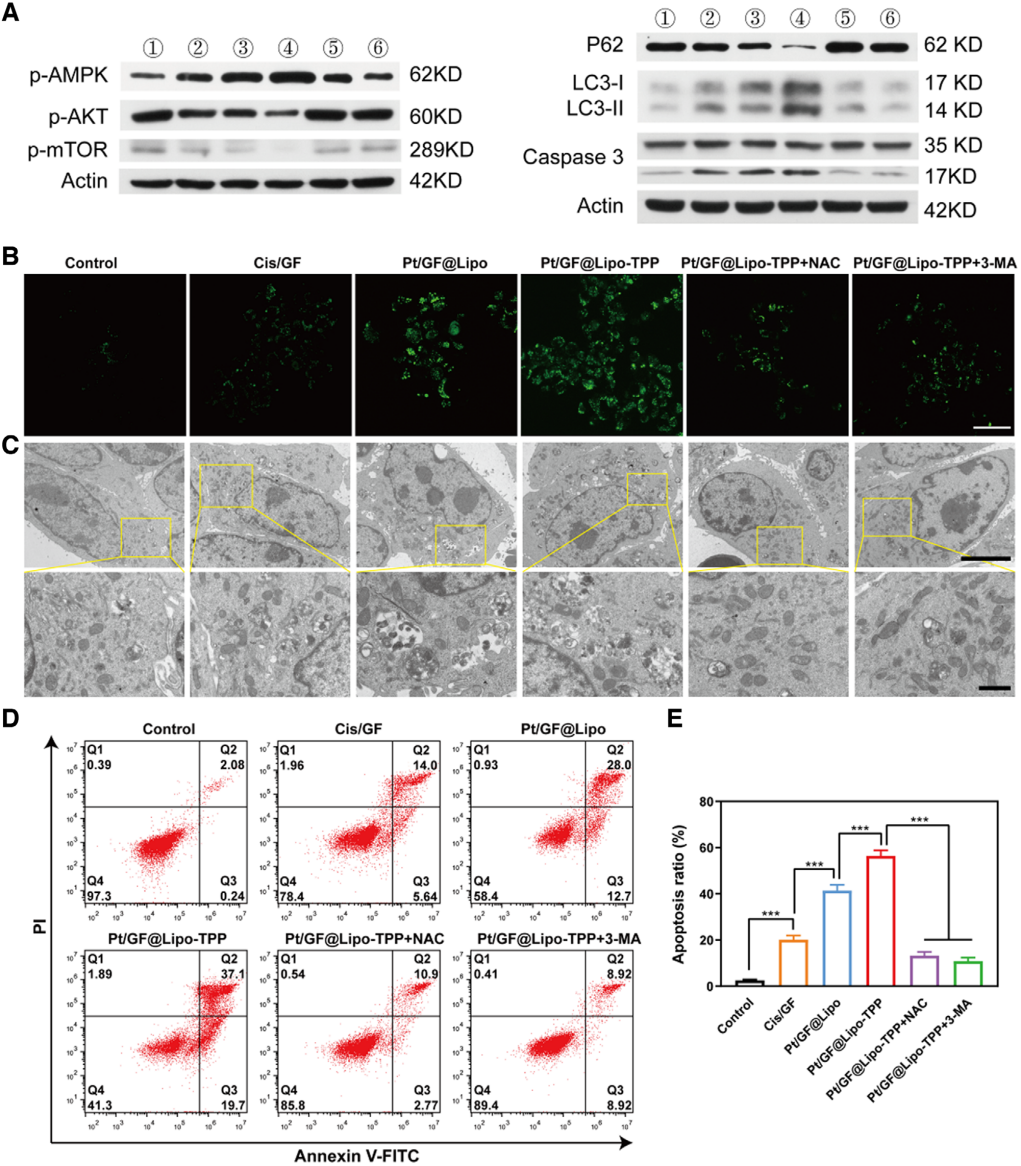
Autophagy induced by Pt/GF@Lipo‐TPP. (A) Western blot analysis of p‐AMPK, p‐AKT, p‐mTOR, P62, LC3 and caspase 3 in 4T1 cells incubated with varied treatment (①: Control, ②: Cis/GF,③: Pt/GF@Lipo, ④: Pt/GF@Lipo‐TPP, ⑤: Pt/GF@Lipo‐TPP+NAC, ⑥: Pt/GF@Lipo‐TPP+3‐MA). (B) CLSM images of DAPGreen stained 4T1 tumor cells cultured with various formulations. Scale bars = 50 μm. (C) Bio‐TEM images of 4T1 tumor cells incubated with different formulations at low (scale bars = 5 μm) and high (scale bars = 1 μm) magnifications. (D,E) Cell apoptosis of 4T1 tumor cells after incubation with varied formulations by flow cytometry analysis with the statistics of cell apoptosis ratio

Autophagy is a common cellular process where cytoplasmic components are generally degraded in the lysosome.^[^ ^15^ ^]^ In recent years, autophagy has emerged as an important mechanism for cell death induced by certain anticancer agents, particularly resulting from the cellular oxidative stress response. The mitochondria‐targeting Mito‐Q was reported to primarily induce autophagy accompanied with cell apoptosis in breast tumor cells through a mechanism involving ROS production.^[^ ^16^ ^]^ Thus, we hypothesize that cell autophagic death is the dominant mechanism by which Pt/GF@Lipo‐TPP ultimately exerts its antitumor effects, while the generation of ROS and the resultant mitochondrial damage are early events related to Pt/GF@Lipo‐TPP. As shown in Figure 5A, Pt/GF@Lipo‐TPP modulates adenosine 5′‐monophosphate (AMP)‐activated protein kinase (AMPK) and protein kinase B (AKT)/mammalian target of rapamycin (mTOR) signaling molecules, which are the autophagy‐associated energy‐sensing proteins.^[^ ^17^ ^]^ To be specific, Pt/GF@Lipo‐TPP elevates the levels of phosphorylated AMPK as well as decreases the phosphorylation of AKT and mTOR, verifying the downregulation of the AKT/mTOR signaling (Figure S13). To further explore the potential mechanism regarding the tumor cell autophagic death induced by Pt/GF@Lipo‐TPP, the lysates of cells incubated with Pt/GF@Lipo‐TPP are analyzed for proteins involved in autophagy inclusive of microtubule‐associated protein light chain 3 (LC3) and sequestosome 1 (P62). By a ubiquitination‐like system, the LC3 precursor could be modified to form LC3‐I which is subsequently modified to LC3‐II as a typical early marker of autophagy. Pt/GF@Lipo‐TPP treatment can significantly elevate the levels of LC3‐II in 4T1 cells (Figure 5A; Figure S13). P62 as a kind of ubiquitin‐binding mitophagy receptors could demonstrate autophagic flux or the completed autophagic degradation procedure as well.^[^ ^18^ ^]^ The levels of P62 in 4T1 cells incubated with Pt/GF@Lipo‐TPP are substantially decreased compared with other groups (Figure 5A; Figure S13).

Such a result was further validated by fluorescent imaging and bio‐TEM measurements. We applied DAPGreen (a fluorescent indicator of autophagosome and early autolysosome) to verify the cell autophagy caused by Pt/GF@Lipo‐TPP. As shown in Figure 5B, the obvious green fluorescent signal in the Pt/GF@Lipo‐TPP group is in accordance with the western blot data. Moreover, the autophagic death that occurred in 4T1 cells has been observed through bio‐TEM. Different from the control group that featured an inherent autophagic process, the quantity of autophagic vacuoles (a typical double‐membrane cytosolic vesicle) is evidently increased in 4T1 cells treated with Pt/GF@Lipo‐TPP, suggesting its induction of cell autophagy. Notably, the induction of autophagy by Pt/GF@Lipo‐TPP treatment can be significantly inhibited by NAC, indicating that the ROS production is the early event associated with cell autophagy induced by Pt/GF@Lipo‐TPP. It is well‐known that autophagy exerts two important and seemingly opposing functions in cancer.^[^ ^19^ ^]^ Autophagy can be either oncogenic or tumor suppressive, which depends on the tumor types and microenvironment.^[^ ^20^ ^]^ Because autophagy induction is capable of promoting tumor cell survival in the condition of nutrient deficiency, massive researches concentrate on exploited autophagy inhibitors to impede cytoprotective autophagy.^[^ ^21^ ^]^ However, upon some environmental stressors, such as ROS production, autophagy induction can give rise to tumor cell death.^[^ ^22^ ^]^ 3‐MA as an autophagy inhibitor was utilized to study whether the cell autophagy induced by Pt/GF@Lipo‐TPP promotes the 4T1 cell death. The cell apoptosis is dramatically increased in cells treated with Pt/GF@Lipo‐TPP, and the apoptosis rate is estimated to be 56.4%. As shown in Figure 5, 3‐MA evidently inhibits the cell autophagy caused by Pt/GF@Lipo‐TPP and decreases the apoptotic rate of 4T1 cells, suggesting that the autophagy induced by Pt/GF@Lipo‐TPP can promote tumor cell death. Taken together, Pt/GF@Lipo‐TPP inhibits tumor cell proliferation by promoting the generation of ROS, stimulating mitochondrial dysfunction, downregulating AKT/mTOR signaling and causing cell autophagic death in breast tumor cells.

### 2.5 In vivo therapeutic performance of Pt/GF@Lipo‐TPP

**FIGURE 6:**
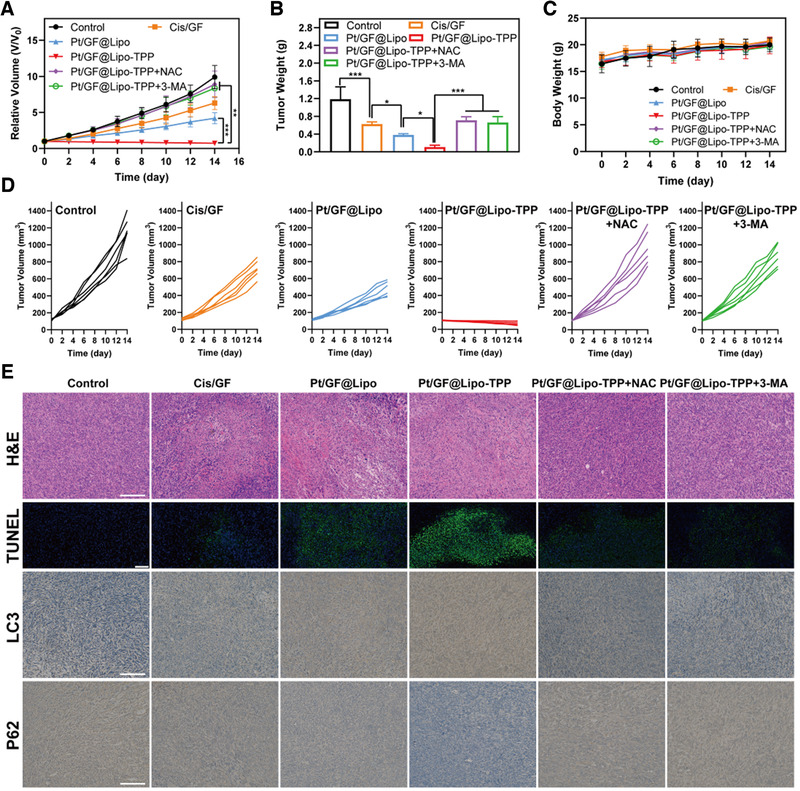
In vivo therapeutic performance of Pt/GF@Lipo‐TPP. (A) Time‐dependent tumor‐growth curves of mice after various treatments. (B) Tumor weights of mice administrated with varied formulations at 14 days. (C) Time‐dependent body‐weight curves within 14 days. (D) Individual tumor growth curves of mice administrated with varied formulations. (E) Tumor sections were stained with H&E, TUNEL, LC3, and P62. Scale bars = 50 μm in H&E, LC3, and P62 images. NAC is a ROS scavenger and 3‐MA is an autophagy inhibitor. Scale bars = 100 μm in TUNEL images

Before investigating the in vivo antitumor efficiency, serial assays were conducted to confirm the biosafety of Pt/GF@Lipo‐TPP in vivo. The results of serum biochemistry, hematology, and histological analysis prove that negligible pathological change or organ damage occurred in mice after intravenous injection of Pt/GF@Lipo‐TPP within 1 month, suggesting its excellent biocompatibility within the administrated dosage (Figures S14–S17). Encouraged by the remarkable chemotherapy‐augmented chemodynamic therapeutic performance in vitro and outstanding biocompatibility in vivo, we then explored the antitumor efficiency of Pt/GF@Lipo‐TPP in vivo. The tumor sizes of all groups were recorded every 2 days to measure and analyze the therapeutic performance (Figure 6A,D). Both NAC (a ROS scavenger) and 3‐MA (an autophagy inhibitor) were applied to construct negative control for illustrating the anticancer mechanism of Pt/GF@Lipo‐TPP in vivo. The chemotherapy‐augmented sequential CDT substantially inhibits tumor progression, indicating the high potential of Pt/GF@Lipo‐TPP for combating tumor (Figure 6B; Figure S18). Additionally, no obvious loss of body weight was observed throughout the whole experimental period, which implies that negligible side effects result from these treatments (Figure 6C). Moreover, both the hematoxylin and eosin (H&E) and TdT‐mediated dUTP‐biotin nick and labeling (TUNEL) further validated the prominent antitumor efficacy of Pt/GF@Lipo‐TPP in vivo (Figure 6E). Finally, we verified whether the antitumor effects of Pt/GF@Lipo‐TPP were induced by cell autophagy in vivo. The expression of LC3, P62, and cleaved‐caspase 3 was in accordance with the in vitro results, implying the autophagy‐mediated anticancer mechanism (Figure 6E).

## 3 CONCLUSIONS

In conclusion, we propose a therapeutic modality of “chemotherapy‐augmented sequential chemoreactive therapy” on the basis of the designed Pt/GF@Lipo‐TPP nanocatalysts, which have exhibited distinct catalytic cancer‐therapeutic performance. This desirable therapeutic outcome was mainly attributed to the specific role of each component in the Pt/GF@Lipo‐TPP nanocatalysts where the bioactive cisplatin converted O_2_ to H_2_O_2_ via the activation of NOX‐related cascaded reaction, providing adequate reactants for the subsequent Fenton catalytic reaction. Meanwhile, the efficient and stable GA‐Fe(II) nanocomposites catalyzed H_2_O_2_ into highly toxic •OH to inhibit tumor cell proliferation. Moreover, the underlying mechanism of antitumor effects regarding Pt/GF@Lipo‐TPP nanocatalysts was chiefly through promoting the generation of ROS, stimulating mitochondrial dysfunction, downregulating AKT/mTOR signaling, and causing cell autophagic death in breast tumor cells. Thus, this work successfully integrates clinical chemotherapy with emerging CDT for dealing with the crucial issues of clinical tumor therapy, paving a promising way for the design and development of efficient modalities with concurrent high anticancer efficiency and mitigated side effects.

## CONFLICT OF INTEREST

The authors declare no conflict of interest.

## ETHICS STATEMENT

The animal experimental procedure was conducted with the approval of ethics by Ethics Committee of Shanghai University.

## Supporting information

SUPPORTING INFORMATION

## Data Availability

The authors declare that all data needed to support the finding of this study are presented in the article and the Supporting Information. Any other data related to this work are available from the corresponding authors upon reasonable request.
