# Supplementary material for: Mitochondria‐specific nanocatalysts for chemotherapy‐augmented sequential chemoreactive tumor therapy
Source: Exploration (Beijing). 2021 Sep 1;1(1):50–60. doi: 10.1002/EXP.20210149 (PMC10291566; doi:10.1002/EXP.20210149)
Supplement: Supplementary file 1 — SUPPORTING INFORMATION [file EXP2-1-50-s001.doc]

Copyright WILEY-VCH Verlag GmbH & Co. KGaA, 69469 Weinheim, Germany, 2021.

Supporting Information

Mitochondria-specific Nanocatalysts for Chemotherapy-augmented Sequential Chemoreactive Tumor Therapy

Hui Huang, Caihong Dong,* Meiqi Chang, Li Ding, Liang Chen, Wei Feng* and Yu Chen*

H. Huang and Prof. Y. Chen

School of Environmental and Chemical Engineering, Shanghai University, Shanghai, 200444, P. R. China.

E-mail: chenyuedu@shu.edu.cn (Y. Chen)

H. Huang, Dr. L. Chen, Dr. W. Feng and Prof. Y. Chen

Materdicine Lab, School of Life Sciences, Shanghai University, Shanghai, 200444, P. R. China.

E-mail: fengw@shu.edu.cn (W. Feng)

Dr. Caihong Dong

Department of Ultrasound, Zhongshan Hospital, Fudan University, and Shanghai Institute of Medical Imaging, Shanghai, 200032, P. R. China.

Email: dong.caihong @zs-hospital.sh.cn (C. Dong)

H. Huang, Dr. M. Chang, Dr. L. Ding and Prof. Y. Chen

State Key Laboratory of High Performance Ceramics and Superfine Microstructure, Shanghai Institute of Ceramics, Chinese Academy of Sciences, Shanghai 200050, P. R. China.

***Experimental Section***

**Materials**

*cis*-Diamineplatinum-(II) dichloride (cisplatin), gallic acid (GA) and polyvinylpyrrolidone (PVP) were obtained from Sigma-Aldrich (Shanghai) Trading Co., Ltd. (Shanghai, China). 1,2-Distearoyl-sn-glycero-3-phosphoethanolamine-N-[amino(polyethylene glycol)] (DSPE-PEG-NH_2_), including DSPE-PEG_2k_-NH_2_ and DSPE-PEG_5k_-NH_2_, was purchased from Ponsure Biotechnology Company (Shanghai, China). N-hydroxysuccinimide (NHS) and N-(3-dimethylaminopropyl)-N’-ethylcarbodiimide hydrochloride (EDC) were purchased from Adamas-beta Inc. (Shanghai, China). (3-carboxypropyl)triphenylphosphonium bromide (TPP-COOH) were obtained from Aladdin Reagent Co., Ltd. (Shanghai, China). Iron dichloride tetrahydrate (FeCl_2_·4H_2_O), cholesterol, and methylene blue trihydrate (MB) were received from Sinopharm Chemical Reagent Co., Ltd. (Shanghai, China). 1,2-dihexadecanoyl-snglycero-3-phosphocholine (DPPC) were purchased from Avanti Lipids Polar, Inc. (Alabama, USA). Propidium iodide (PI) and calcein acetoxymethyl ester (calcein AM) were obtained from Shanghai Yeasen Biotechnology Co., Ltd. (Shanghai, China). Cell Counting Kit-8 (CCK-8), Hoechst, 2’, 7’-Dichlorofluorescin diacetate (DCFH-DA), Mitotracker green, 5,5,6,6-tetrachloro-1,1,3,3-tetraethylbenzimidazolylcarbocyanine iodide (JC-1) mitochondrial membrane potential probe and fluorescein isothiocyanate (FITC)-conjugated Annexin V (Annexin V-FITC) Apoptosis Detection kits were purchased from Beyotime Institute of Biotechnology (Haimen, Jiangsu, China). Fetal bovine serum (FBS), penicillin/treptomycin and trypsin were purchased from Gibco Life Technologies Co., Ltd. (Grand Island, USA). Roswell Park Memorial Institute (RPMI) 1640 medium was acquired from Hyclone Laboratories (Logan, Utah, USA). Paraformaldehyde was purchased from Beijing Dingguo Changsheng Biotechnology Co., Ltd. (Beijing, China). All other chemicals and reagents were of analytical grade and used as received from Sigma-Aldrich (Shanghai) Trading Co., Ltd. (Shanghai, China) without further purification.

**Synthesis of DSPE-PEG_2k_-Pt(IV)**

*c,c,t*-[Pt(NH_3_)_2_Cl_2_(OH)_2_] and *c,c,t*-[Pt(NH_3_)_2_Cl_2_(OOCCH_2_CH_2_COOH)(OH)] (Pt(IV)) were synthesized by following the procedure described by *S Dhar et al*.^[1]^ The DSPE-­PEG_2k_-NH_2_ was functionalized with Pt(IV) *via* an amide reaction between the amino groups of DSPE-PEG_2k_-NH_2_ and the carboxyl groups of Pt(IV) in the presence of EDC/NHS. Briefly, Pt(IV) (43.2 mg, 0.1 mmol), NHS (46 mg, 0.4 mmol) and EDC (76.4 mg, 0.4 mmol) were dissolved in DMSO (2 mL). After stirring for 30 min, a DMSO solution of DSPE-PEG_2k_-NH_2_ (274 mg, 0.1 mmol, 1 mL) was added to the mixed solution. The reaction mixture was kept stirring at room temperature for 72 h and then dialyzed in a Slide-A-Lyzer dialysis cassette (Thermo Scientific, Shanghai, China) (1000 Da molecular weight cutoff) against water. The dialysate containing the pure product was lyophilized, and the residue was dried in vacuo over P_2_O_5_.

**Synthesis of DSPE-PEG_5k_-TPP**

Briefly, TPP-COOH (107.3 mg, 0.25 mmol), EDC (191 mg, 1 mmol) and NHS (115 mg, 1 mmol) were dissolved in dimethyl sulfoxide (DMSO, 3 mL). After 30 min stirring, DSPE-PEG_5k_-NH_2_ (1435 mg, 0.25 mmol) DMSO solution (2 mL) was added to the activated TPP complex solution. Subsequently, the reaction mixture was kept stirring for 24 h and then dialyzed in a Slide-A-Lyzer dialysis cassette (Thermo Scientific, Shanghai, China) (3500 Da molecular weight cutoff) against water at room temperature. The dialysate containing the pure product was lyophilized, and the residue was dried in vacuo over phosphorus pentoxide (P_2_O_5_).

**Preparation of GA-Fe(II) Nanocomposites**

GA-Fe(II) nanocomposites were prepared according to the previously described procedure with minor modifications.^[2]^ Briefly, PVP (160 mg) was mixed with FeCl_2_·4H_2_O (46 mg) in 8 mL of degassed deionized (DI) water and allowed to vigorously stir for 5 min at room temperature. Afterthat, GA (2 mL, 10 mg mL^–1^) degassed aqueous solution was dropwise added to above mentioned reaction mixture and then stirred under nitrogen protection for 24 h. Finally, the fabricated GA-Fe(II) nanoscomposites with purple color were harvested and purified by utilizing ultrafiltration filters (10 kDa molecular weight cutoff), and then the obtained samples were stored at 4 °C for further use.

**Fabrication of Pt/GF@Lipo-TPP Nanocatalysts**

The dried lipid film was fabricated by following a reported process with some modification.^[3]^ Briefly, DSPE-PEG_5k_-TPP, DSPE-PEG_5k_, DSPE-PEG_2k_-Pt(IV), DPPC and cholesterol were dissolved in 1 mL dichloromethane and then dired in the vacuum to form the lipid films. Afterward, Pt/GF@Lipo-TPP nanocatalysts were synthesized by lipid films hydration with GA-Fe(II) nanocomposite aqueous dispersions, extruded through a polycarbonate membrane with 200 nm pore size, and purified by running sephadex G-100 columns. The obtained Pt/GF@Lipo-TPP nanocatalysts were further condensed and stored at 4 °C.

**Characterization**

Transmission electron microscopy (TEM) images of the samples were carried out for the morphology observation on a JEM-2100F field emission transmission electron microscope (JEOL, Japan). The hydrodynamic sizes and Zeta-potential of the samples werecharacterized *via* dynamic light scattering (DLS) in a Nano ZS 960 Malvern Zetasizer Nanoseries (Malvern Instrument Ltd. UK). The confocal fluorescence imaging was acquired through an FV1000 confocal microscope (Olympus Company, Japan). Ultraviolet-visible-near-infrared (UV-vis-NIR) absorption spectra were measured on a Shimadzu UV-3600 UV-vis-NIR scanning spectrometer (Shimadzu Scientific Instruments, Japan). The concentration of Pt and Fe elements was determined by inductively coupled plasma optical emission spectrometry (ICP-OES) on an Agilent 725 ICP-OES System (Agilent Technologies, US).

**Assessment of Hydroxyl Radicals (•OH) Generation**

MB degradation reaction was employed to detect the generation of •OH in the Fenton-like reaction. Briefly, 5 mL of phosphate buffer saline solution (PBS, pH = 6.5) containing GA-Fe(II) (dose: 10 μg mL^-1^), H_2_O_2_ (1 mM) and MB (15 μg mL^–1^) was incubated at 37 °C for various time intervals, and then the absorbance of the solutions was measured by using UV-vis-NIR scanning spectrometer at 665 nm to record the degradation of MB.

The generation of hydroxyl free radicals (•OH) and elecron paramagnetic resonance (EPR) spectra was further carried out on an A200S-95/12 electron paramagnetic resonance spectrometer (Bruker, Germany) using 5,5-dimethyl-l-pyrroline-N-oxide (DMPO) as the spin trapping agent.

**Cell Culture**

4T1 mouse breast tumor cell line was purchased from the Cell Bank of Shanghai Institutes for Biological Sciences, Chinese Academy of Sciences (Shanghai, China), and then cultured in RPMI-1640 medium supplement with 10% FBS, penicillin (100 units/mL) and streptomycin (100 μg/mL). All cultures were kept at a humidified atmosphere containing 5% CO_2_ at 37 °C. The media were replaced every two days, and the cells were passaged by trypsinization before approached to 80% confluence.

**Cellular Uptake**

To confirm the intracellular phagocytosis of Pt/GF@Lipo-TPP, the FITC labelled Pt/GF@Lipo-TPP was prepared. After that, 4T1 cells (5×10^3^ cells/well) were cultured in confocal dish and subsequently incubated with RPMI-1640 medium containing free FITC, FITC@Lipo and FITC@Lipo-TPP (dose: 200 μg mL^-1^) for 1, 4 and 8 h, respectively. After co-incubation, the cells were washed gently 3 times with PBS to remove redundant samples and collected for subsequent flow cytometry (FCM) analysis and confocal laser scanning microscopy (CLSM) imaging.

**Determination of Pt and Fe in 4T1 Cells by ICP-OES**

4T1 cells (3×10^5^ cells per well) were seeded in six-well culture plates at 37 °C for 12 h. After that, the cells were treated with culture medium (2 mL) containing free drug, Pt/GF@Lipo, and Pt/GF@Lipo-TPP with the platinum concentration regulated to the same value of 10 μM for 1, 4 and 8 h respectively. After treatment, the cells were thoroughly washed with PBS thrice, and then lysed by using aqua regia. The contents of Pt and Fe in the cell lysis solution were measured by ICP-OES and the cells without sample treatments was selected as blank controls.

**Cellular Uptake Mechanisms**

To study the mechanism of cellular uptake of Pt/GF@Lipo-TPP in 4T1 cells, the cells (5×10^3^ cells/well) were seeded in glass-bottom cell culture dishes and six-well culture plates, and then pre-incubated with different endocytosis inhibitors including 5 μM chlorpromazine (Chl), 5 mM amiloride (Ami), and 10 μM nystatin (Nys) at 37 °C for 1 h. After that, FITC@Lipo-TPP (dose: 200 μg mL^-1^) was added into each dish and incubated for 4 h. Then the cells were imaged by the CLSM imaging system and collected for flow cytometry analysis.

**Colocalization Analysis with Mitochondria**

CLSM imaging was performed to observe the co-localization of Pt/GF@Lipo-TPP with mitochondria. Briefly, 4T1 cells (1 × 10^3^ cells) were seeded in glass-bottom cell culture dishes and incubated for 24 h. Then, the medium was replaced with fresh RPMI 1640 medium containing Rh B@Lipo-TPP (dose: 20 μg mL^-1^) for 4 h. Afterthat, cells were washed twice with cold PBS and then incubated with Mitotracker green (100 nM) to label mitochondria according to the manufacturer’s protocol, respectively. Then cells were imaged by the CLSM imaging system with identical settings.

***In Vitro* Cytotoxicity Assay**

The cytotoxicity of different formulations was investigated by examining the 4T1 cells viabilities using the CCK-8 assay. Briefly, 4T1 cells (8×10^3^ cells per well) were seeded into 96-well plates and cultured 24 h to allow cells to attach. After that, the old medium was replaced with fresh medium as blank control and the medium containing GF@Lipo, cisplatin, Pt@Lipo, Cis/GF, Pt/GF@Lipo and Pt/GF@Lipo-TPP with various concentrations were added into the plates. After exposure for another 24 and 48 h at 37 °C, the supernatant was removed and then rinsed twice carefully with PBS, followed by the addition of RPMI 1640 media (100 μl) and CCK-8 (10 μL). After incubation for another 2 h, the cell viability was analyzed by measuring the absorbance at 450 nm by using a Spectra M2 plate reader (Molecular Devices, CA, USA). The relative cell viability was expressed as:


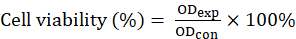


Where *OD_exp_* is the OD value of the experimental sample, *OD_con_* is the OD value of the control group, and the average value was obtained from five parallel samples.

**Calcein-AM/PI Assay**

The 4T1 cells (1×10^5^ cells/well) were seeded in glass-bottom cell culture dish overnight and treated with 2 mL of GF@Lipo, cisplatin, Pt@Lipo, Cis/GF, Pt/GF@Lipo and Pt/GF@Lipo-TPP at a Pt dose of 10 µM for 24 and 48 h. Nontreated 4T1 cells were used as blank control. After washing with PBS three times, 1 mL mixed solution containing Calcein-AM (2 μM) and PI (500 nM) was added into each dish, and the ﬂuorescent images were immediately acquired using CLSM.

**Intracellular Reactive Oxygen Species (ROS) Detection**

The intracellular reactive oxygen species (ROS) generation after various treatments was explored using 2’,7’-dichlorodihydrofluorescin diacetate (DCFH-DA) assay. DCFH-DA, a non-fluorescent cell-permeable compound for the ROS detection, diffuses into cells and forms 2,7’-dichlorodihydrofluorescin (DCFH) after cleavage of the two acetate groups by intracellular esterases. After that, ROS induced DCFH oxidation results in generation of the highly fluorescent product 2’,7’-dichlorofluorescein (DCF). For confocal microscopy observation, 4T1 cells (10^5^ cells per dish) were plated onto 20 mm glass-bottom cell culture dishes and incubated 24 h for cell attachment. After that, the medium was removed and added serum free medium supplement with DCFH-DA (10 μM) at 37 °C for 30 min. After removing the dye, the dishes were washed three times with medium, and the cells were then incubated with RPMI 1640 media (1 ml) containing Cis/GF, Pt/GF@Lipo, Pt/GF@Lipo-TPP and Pt/GF@Lipo-TPP+NAC with the platinum concentration regulated to the same value of 10 μM at 37 °C for 4 h. Finally, cells were rinsed and fluorescence visualization was performed by a CLSM, in which the excitation wavelength was at 488 nm and the emission peak was examined at 525 nm.

**Mitochondrial Membrane Potential**

4T1 cells (5×10^4^ cells/well) were incubated with different formulations with the platinum concentration regulated to the same value of 10 μM, then incubated with culture medium containing JC-1 dye (1.0 μg ml^−1^) of at 37 °C for 15 min. Then, the cells were washed with PBS, incubated with fresh medium and visualized on a CLSM. The excitation wavelength of JC-1 dyes was at 488 nm and the emission were detected separately at 530 nm for JC-1 monomers and at 585 nm for JC-1 aggregates.

**Bio-TEM for Detecting Mitochondria Damage and Autophagy**

4T1 cells (5×10^4^ cells/well) were incubated with differen formulations with the platinum concentration in culture medium regulated to the same value of 10 μM for 24 h. After that, 4T1 cells were washed with PBS, fixed in osmic acid, sectioned (70 nm) and observed under TEM (HITACHI, HT7700).

**Western Blot Analysis**

Total proteins were collected from 4T1 cells after different treatments and their concentrations were measured by using the BCA protein assay kit. The equal amounts of proteins from each sample (50 μg) were separated by d by sodium dodecyl sulfate polyacrylamide gel electrophoresis (SDS-PAGE), and then transferred to an nitrocellulose (NC) membrane and incubated with anti-P62 (CST, 1:1,500), anti-LC3 (CST, 1:1,500), anti-caspase 3 (CST, 1:1,500), anti-p-AMPK (CST, 1:1,500), anti-p-AKT (CST, 1:1,500) and anti-p-mTOR (CST, 1:1,500) antibodies for 1 h and secondary antibody (1:6000, zsBio) for another 1 h at room temperature.

**Cell Apoptosis**

The apoptosis was analyzed using the Annexin V-FITC apoptosis detection kit and analyzed by flow cytometry. Briefly, the 4T1 cells (5×10^5^ cells/well) were seeded in a 6-well culture plate at 37 °C for 12 h and treated with different formulations for 24 h. The control experiments were performed by adding only culture medium. The collected cell pellets were washed twice with cold PBS, and the cell suspension (1×10^6^ cells/mL) was stained with 5 µL of Annexin V-FITC and PI, vortexed and incubated for 10 min in dark. After staining, the population of apoptotic and non-apoptotic cells were quantified and analyzed by using flow cytometry.

**Animals and Treatment**

All the experimental healthy female Kunming mice (6-7-week old) and female Balb/c nude mice (5-6-week old) were obtained from Shanghai Slac Laboratory Animal Co., Ltd. (Shanghai, China). The animal experimental procedure was conducted with the approval of ethics by Ethics Committee of Shanghai University. All animals were kept individually in ventilated cages with a balanced diet and water ad libitum under controlled conditions of 22 ± 3 °C temperature, (50-60% relative humidity and 12 h light/dark cycle. Animals were acclimatized to the laboratory conditions for seven days prior to the initiation of dosing.

***In Vivo* Bio-toxicity Study**

Female Kunming mice were randomly assigned to two groups for the toxicity assessment (12 active mice in each group and 4 backup ones). All groups were intravenously injected once a week. For the Group 1 as control group, physiological saline acted as blank vehicle was used. In addition, Group 2 was administrated Pt/GF@Lipo-TPP at Pt dose of 2 mg/kg.

The body weight for each animal was measured once every two days, while the hematological and blood biochemical indexes was detected once a week. Blood samples, approximately 1 mL/sample were collected *via* orbital puncture from each animal. The hematological parameters were determined by using an automated hematology analyzing machine. The serum was collected by the centrifugation of the whole blood at 4000 rpm for 15 min. After that, the serum biochemical parameters including alanine aminotransferase (ALT), aspartate aminotransferase (AST), albumin (ALB), alkaline phosphatase (ALP), creatinine (CERA) and carbamide (UREA) were carried out by a Hitachi 7020 automatic biochemical analyzer.

**Histological Examination**

The tissues including heart, liver, spleen, lung and kidney tissues were harvested and collected from each group once a week postinjection. After wash three times with PBS, each tissue was immediately immersed into 4% paraformaldehyde solution, embedded in paraffin, sectioned at five micrometer thickness, stained with hematoxylin and eosin (H&E) and finally observed under a light microscope.

**Tumor Modeling and *In Vivo* Cancer Therapy**

For *in vivo* cancer therapy, 100 μL of 4T1 cells suspension (~10^6^ cell per mouse) were subcutaneously inoculated into the right rear flank of each mouse to establish tumor-bearing model. When the tumor became palpable and the volume reach ~100 mm^3^, the 4T1 tumor-bearing mice were randomly divided into 6 groups (6 mice per group): Group 1 was intravenously administered with saline solution as control group; Group 2-6 were intravenously injected with Cis/GF, Pt/GF@Lipo, Pt/GF@Lipo-TPP, Pt/GF@Lipo-TPP+NAC and Pt/GF@Lipo-TPP+3-MA, respectively. The tumor volumes were monitored for 14 days and conducted using a digital Vernier caliper every 2 days. The tumor volume was calculated according to the following equation:


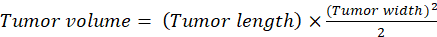


In order to reflect the mice living status, the mcie weights were monitored every two days. After 14 days observation, the tumor were collected and then fixed in 4% paraformaldehyde. Thereafter, the tumor tissues were processed into paraffin and sliced to H&E staining, TUNEL immunofluorescent staining as well as LC3 and P62 immumohistochemical staining.

**Statistical Analysis**

All quantitative values were presented as mean ± standard deviation (SD). The statistical significance of differences among groups were carried out by using one-way analysis of variance (ANOVA) analysis followed by Tukey’s post-test. The statistical significance for the tests was at **P* < 0.05, ***P* < 0.01 and ****P*< 0.005, NS indicates P > 0.05.

***Supplementary figures***


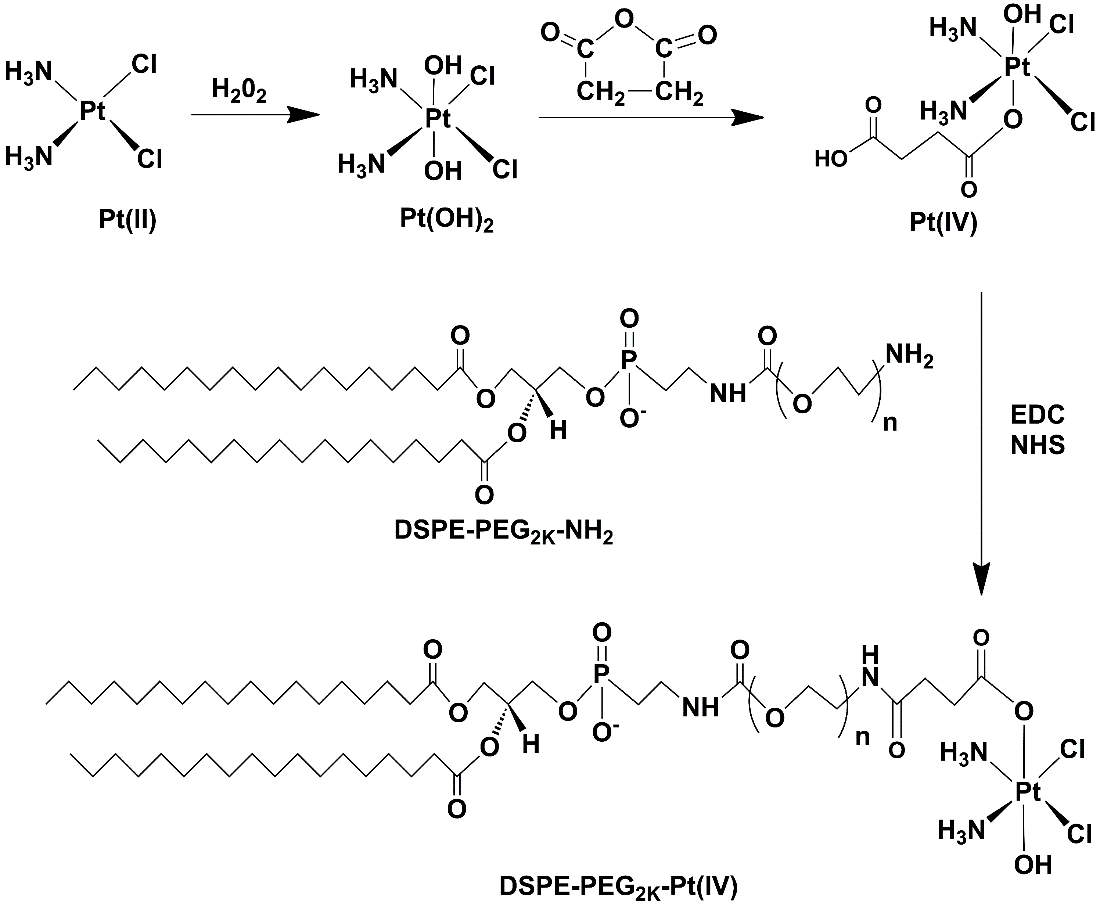


**Figure S1.** The synthetic procedure of DSPE-PEG2k-Pt(IV).


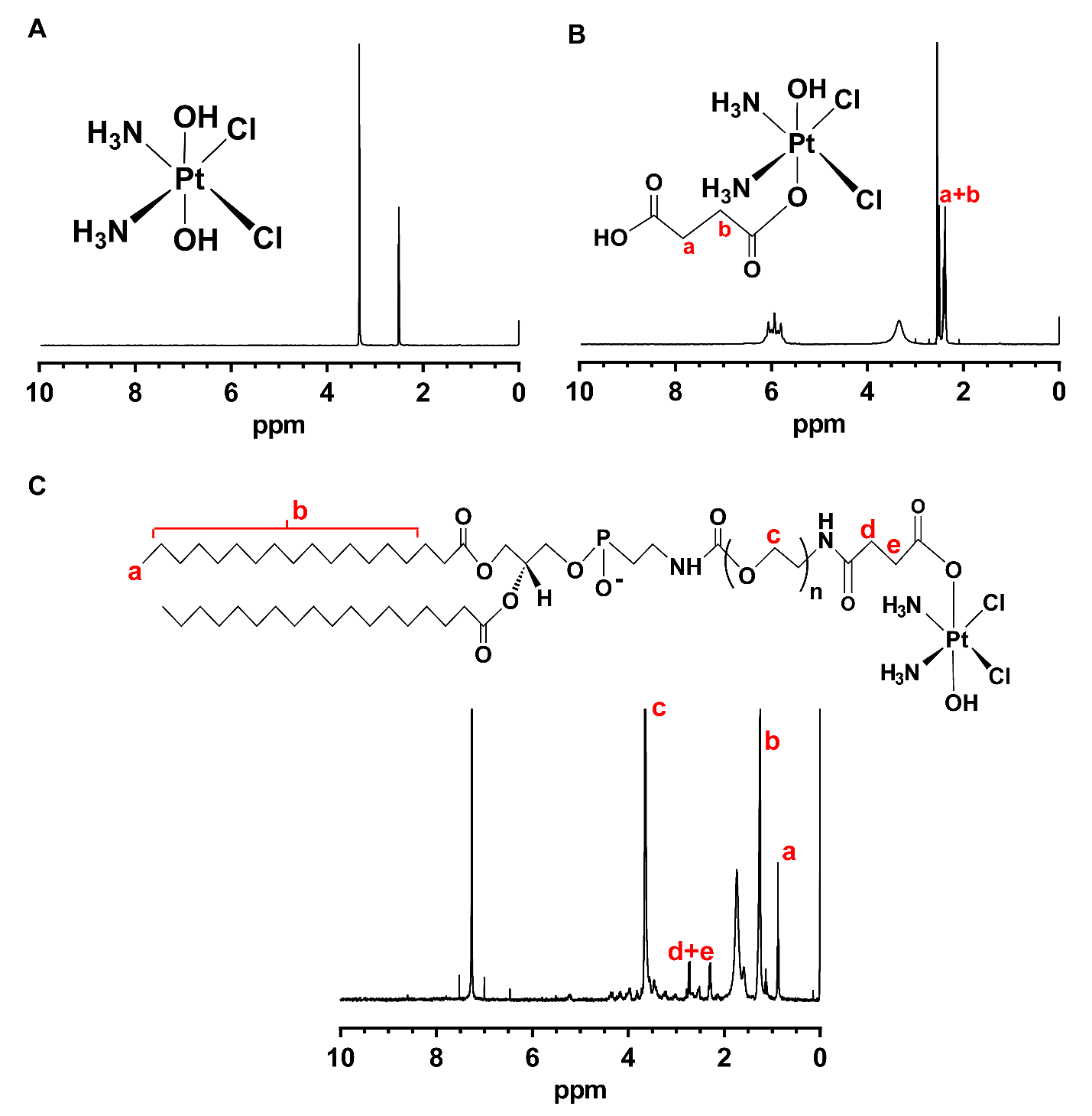


**Figure S2.** ^1^H NMR spectra of *c,c,t*-[Pt(NH_3_)_2_Cl_2_(OH)_2_] (A), *c,c,t*-[Pt(NH_3_)_2_Cl_2_(OOCCH_2_CH_2_ COOH)(OH)] (Pt(IV)) (B) and DSPE-PEG_2k_-Pt(IV) (C).


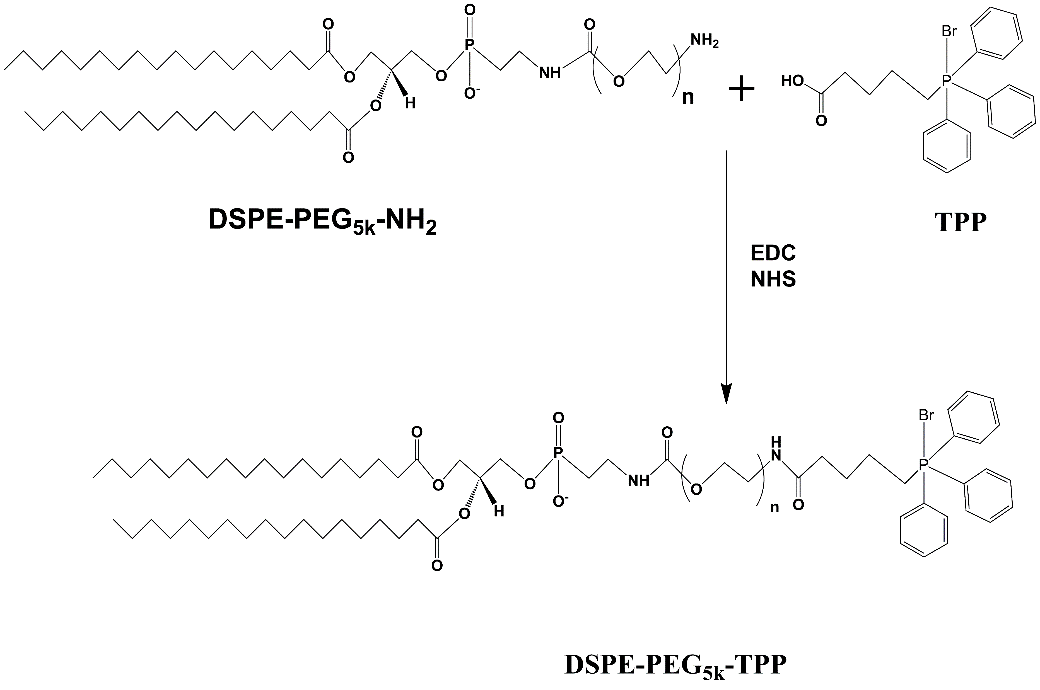


**Figure S3**. The synthetic route of DSPE-PEG5k-TPP.


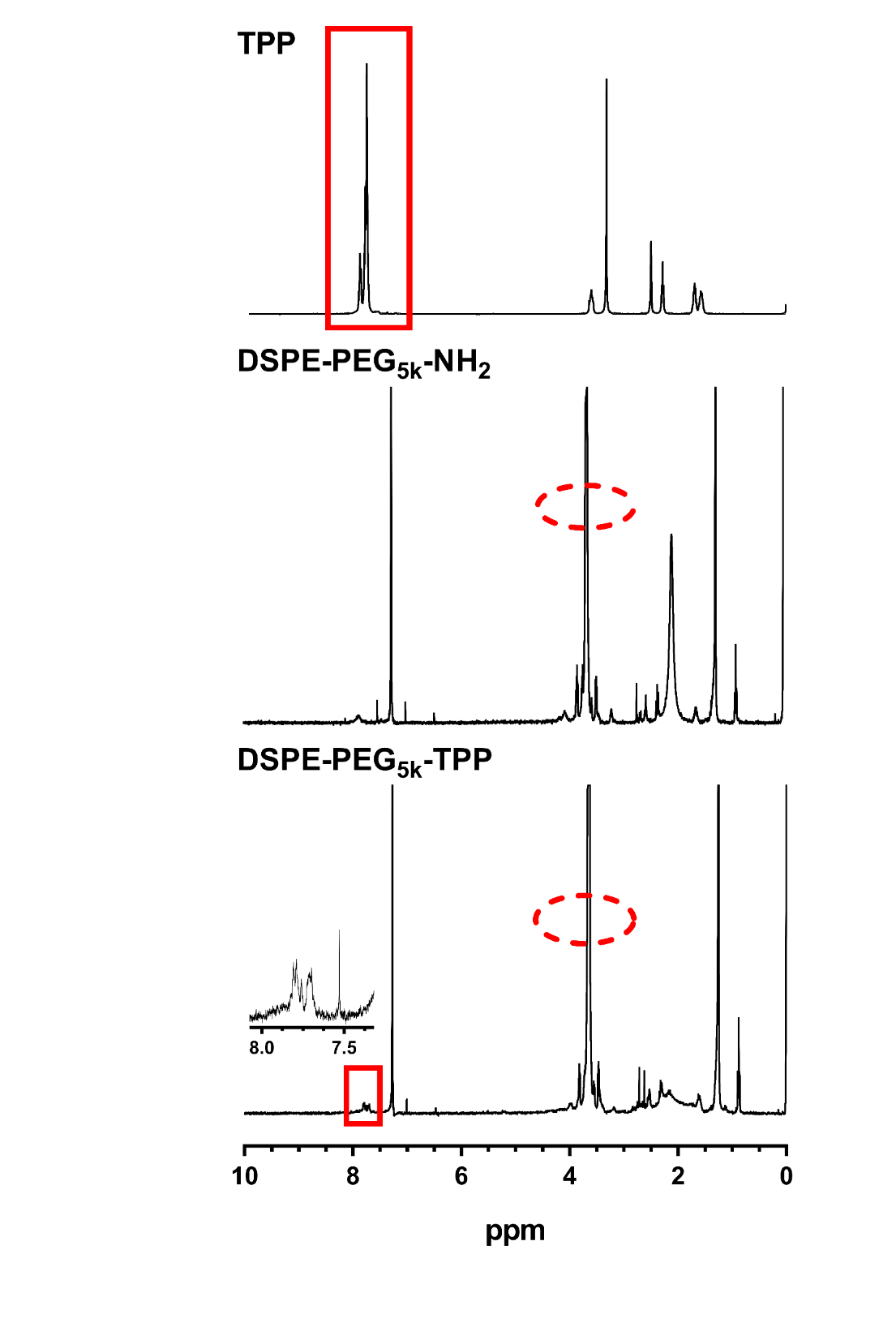


**Figure S4**. ^1^H NMR spectra of TPP, DSPE-PEG_5k_-NH_2_ and DSPE-PEG_5k_-TPP.


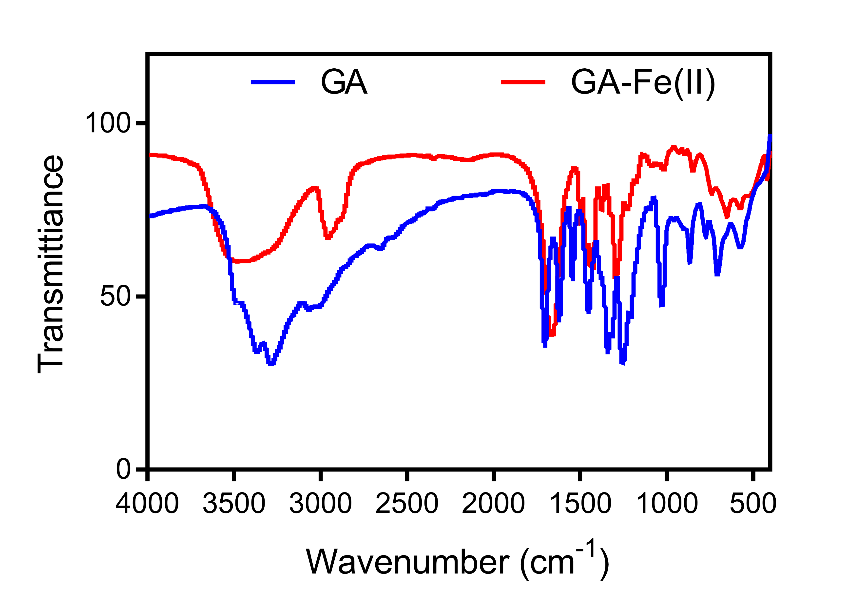


**Figure S5.** FTIR spectra of GA and GA-Fe(II) nanocomposites.


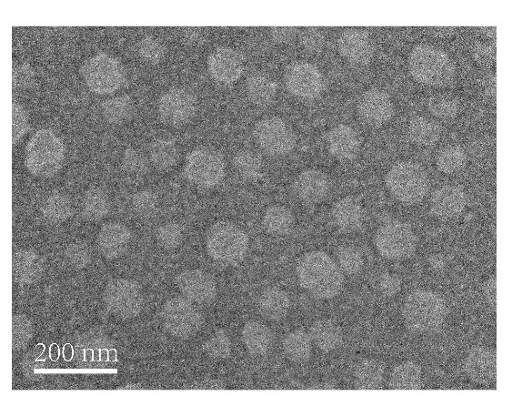


**Figure S6.** TEM image of Pt/GF@Lipo-TPP.


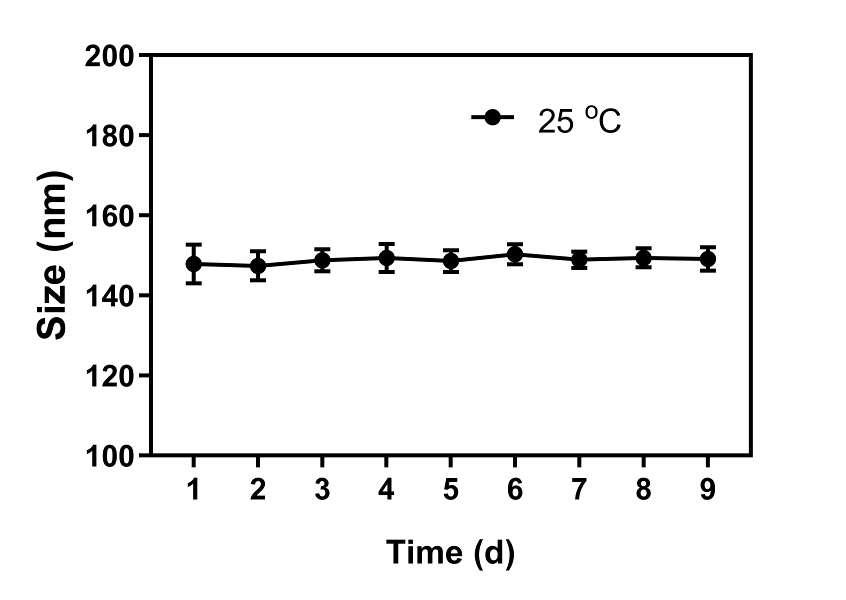


**Figure S7.** Size change of Pt/GF@Lipo-TPP at 25°C.


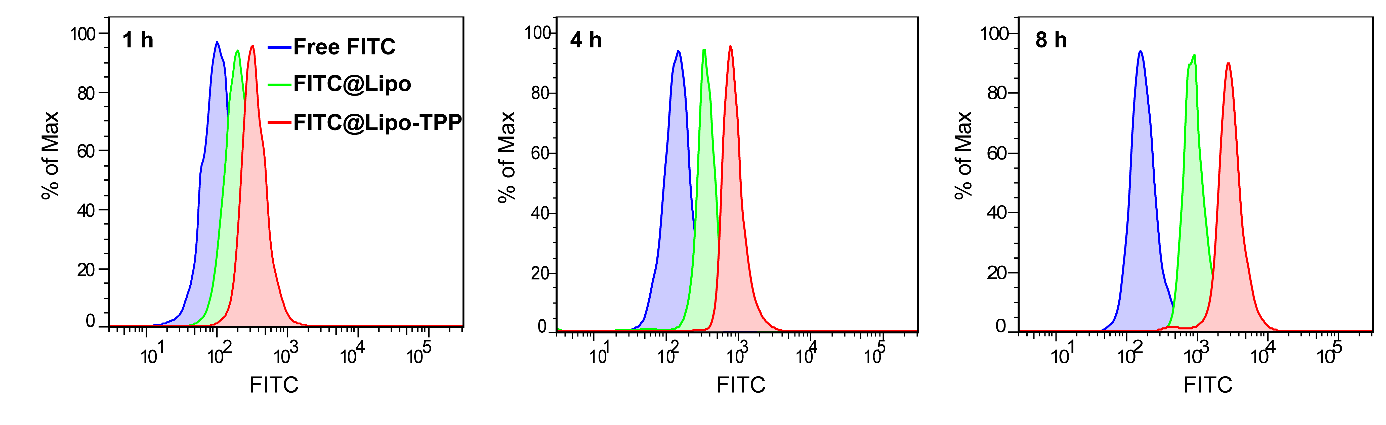


**Figure S8.** The cellular uptake measured by flow cytometer analysis of 4T1 cells treated with free FITC, FITC@Lipo and FITC@Lipo-TPP for 1, 4 and 8 h, respectively.


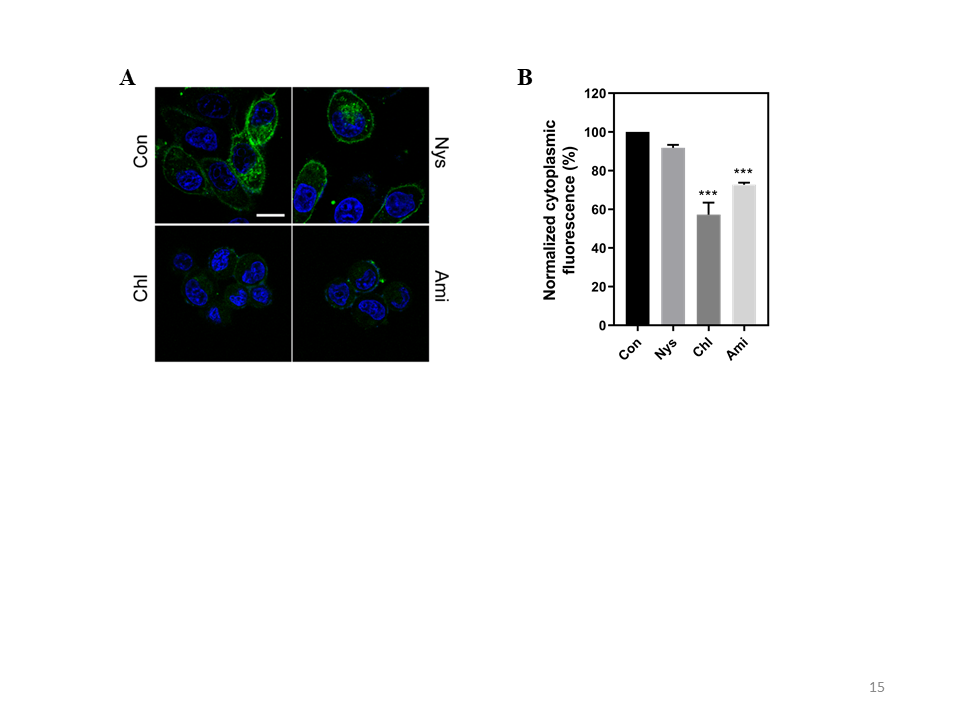


**Figure S9.** (A) CLSM images of 4T1 cells pretreated with different endocytic inhibitors including Nys, Chl and Ami. (B) The corresponding normalized cytoplasmic fluorescent intensity in Figure 3d.


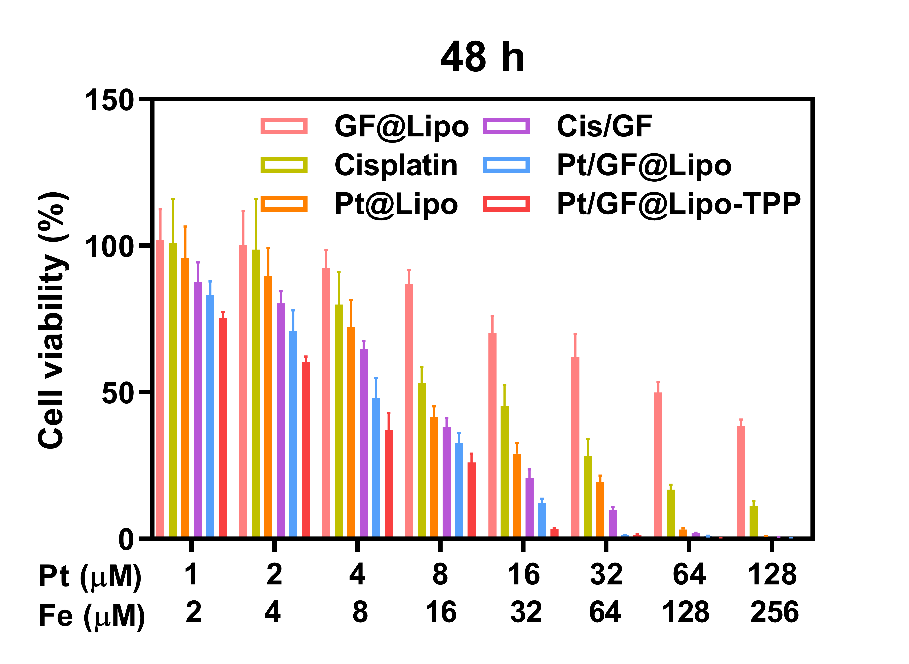


**Figure S10.** Cell viability of 4T1 cells after treatment with various formulations for 48 h.


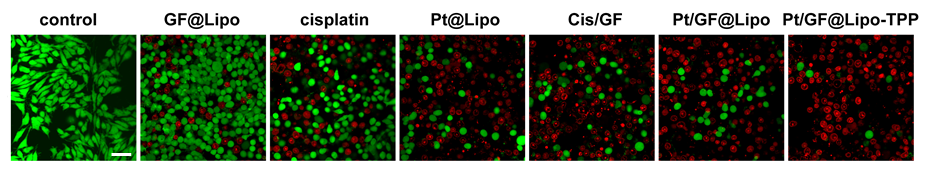


**Figure S11.** CLSM images of 4T1 cells stained with calcein-AM/PI for 48 h. Scale bars = 50 µm.


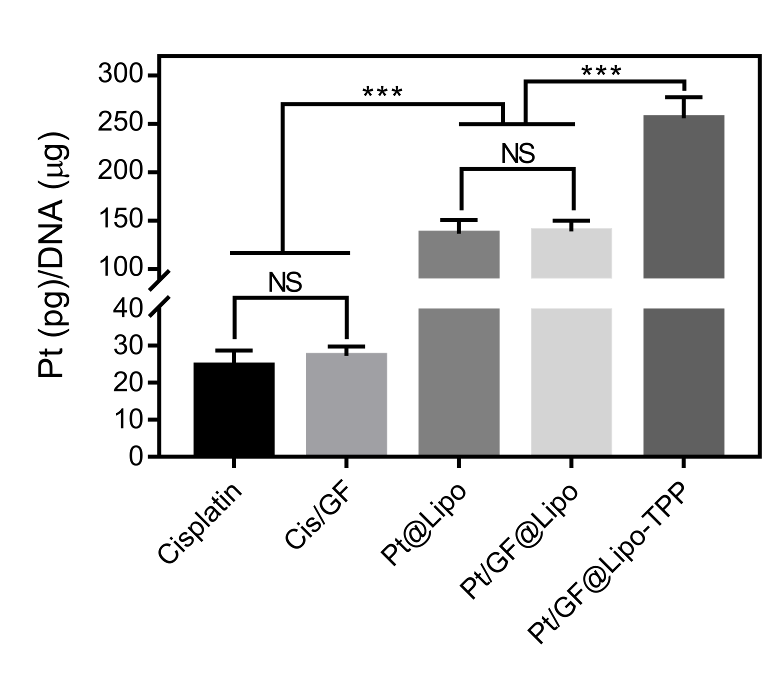


**Figure S12.** Intracellular Pt-DNA adducts after cells incubated with different formulations.

**
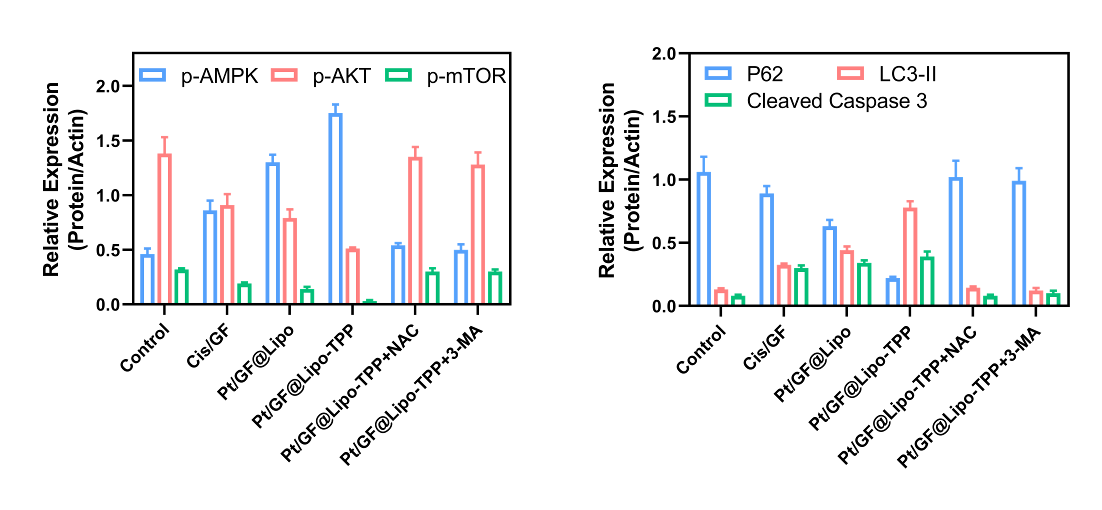
**

**Figure S13.** The corresponding quantitative analysis of protein expression variation after treatment with various groups on basis of western blot results in Figure 5.


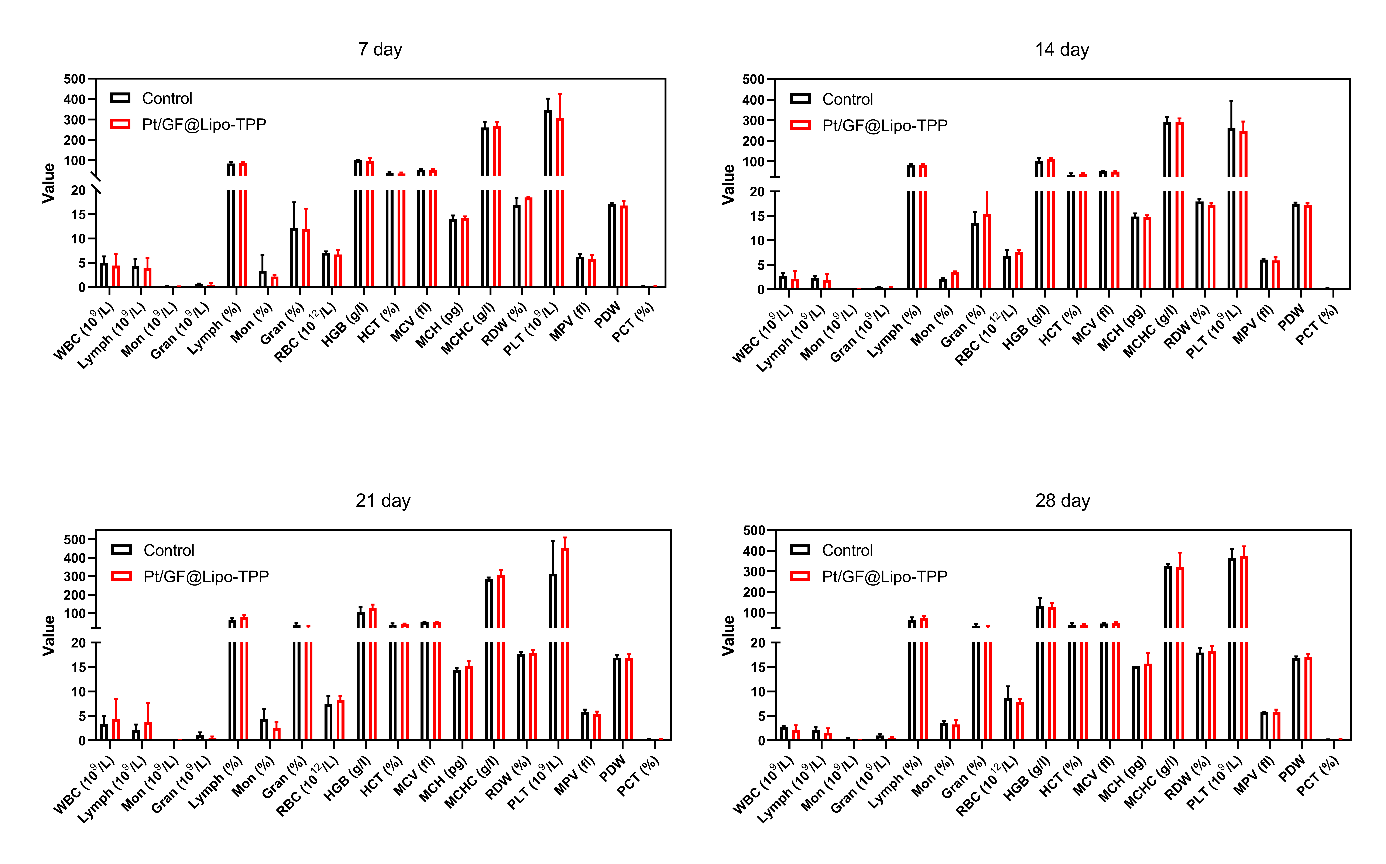


**Figure S14.** Hematological index of the mice with intravenous injection of Pt/GF@Lipo-TPP in 7, 14, 21 and 28 days post-injection.


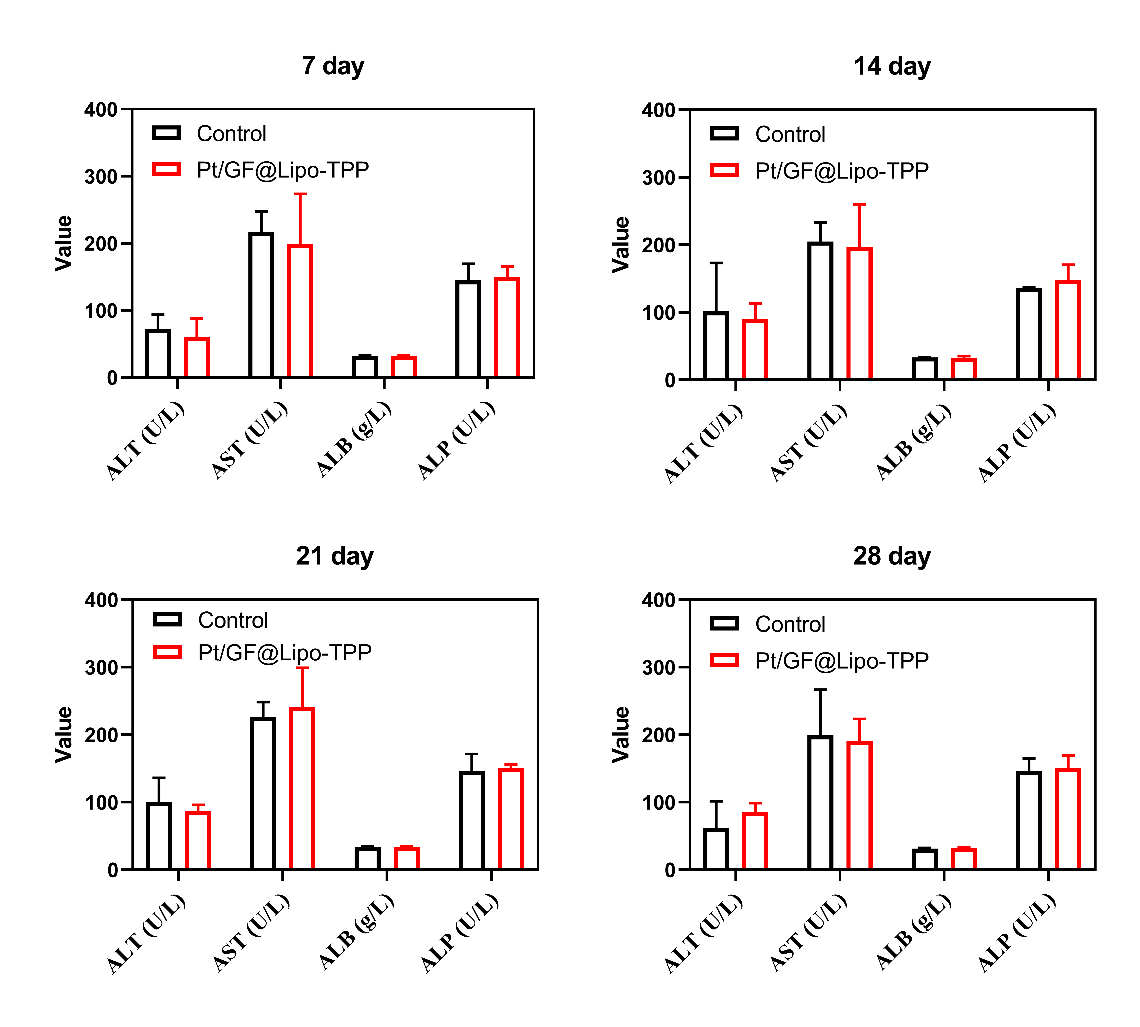


**Figure S15.** Serum ALT, AST, ALB and ALP levels of the mice with intravenous injection of Pt/GF@Lipo-TPP in 7, 14, 21 and 28 days post-injection.


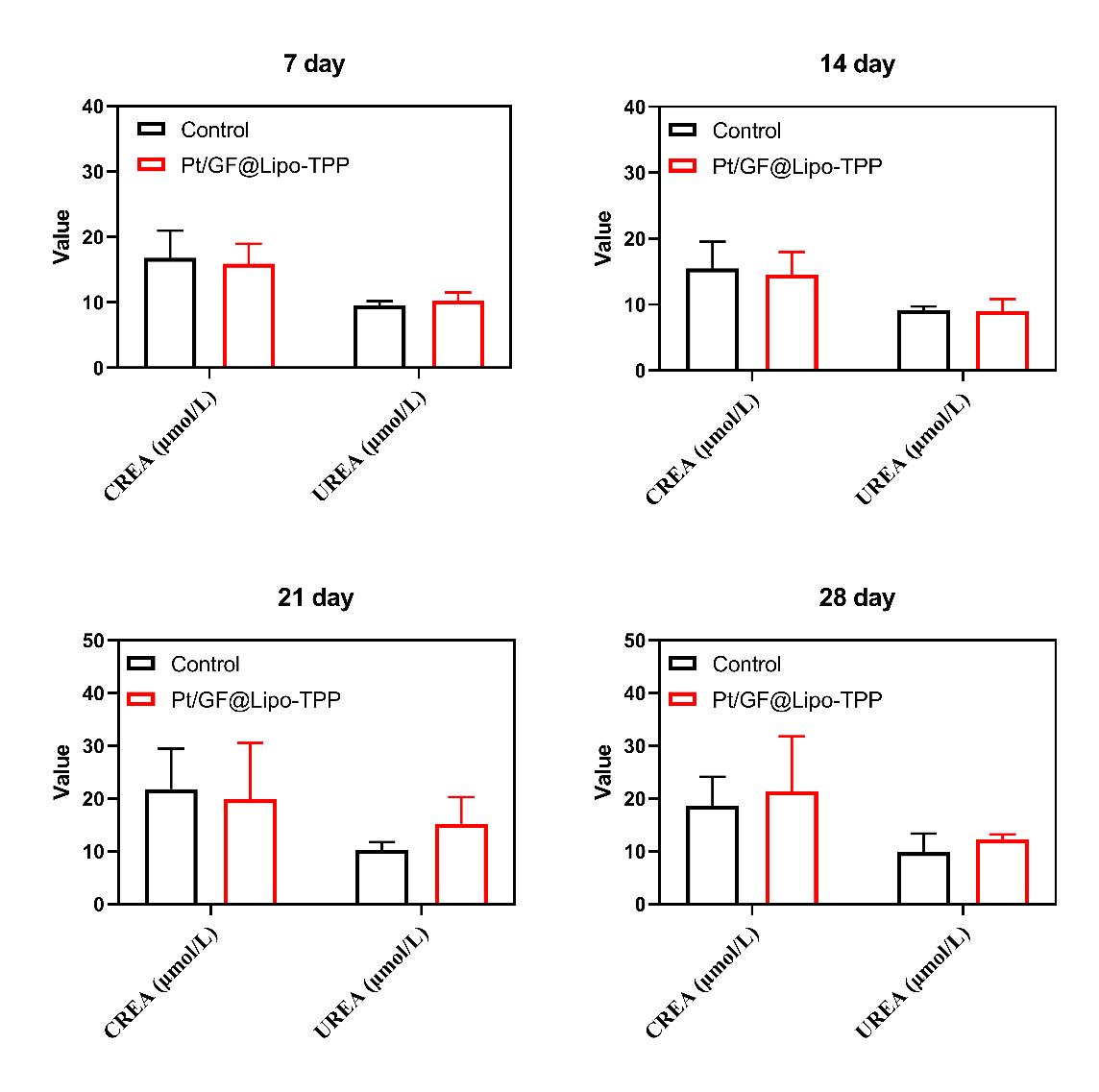


**Figure S16.** Serum CREA and UREA levels of the mice with intravenous injection of Pt/GF@Lipo-TPP in 7, 14, 21 and 28 days post-injection.


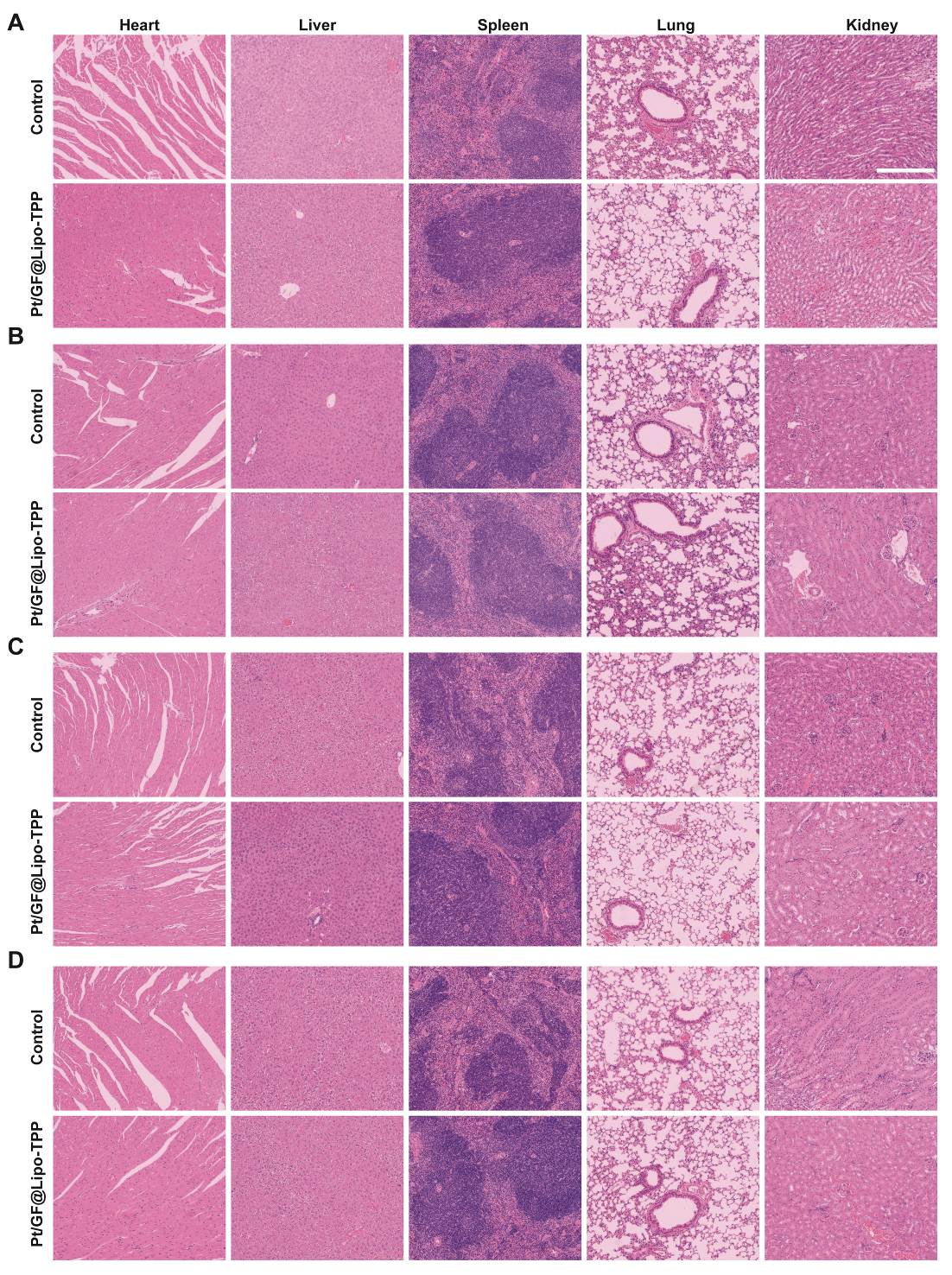


**Figure S17.** H&E staining of the major organs (heart, liver, spleen, lung and kidney) of the mice treated with Pt/GF@Lipo-TPP in 7 (A), 14 (B), 21 (C) and 28 (D) days post-injection. Scale bars = 200 µm.


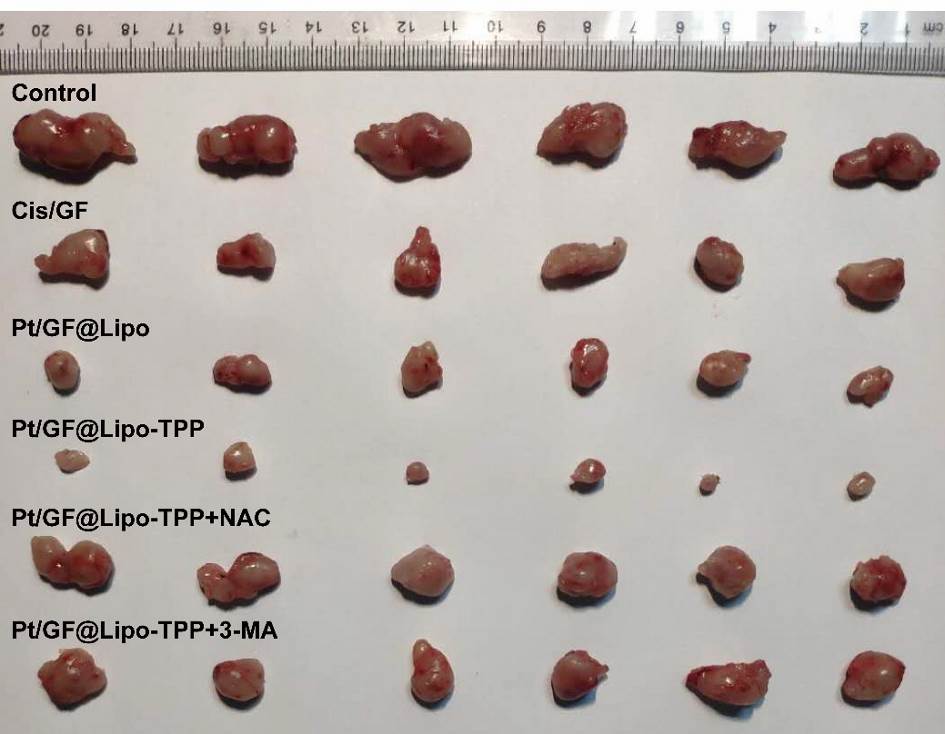


**Figure S18.** The representative digital photo of the tumor dissected from various groups.

**References:**

[1] Y. Yang, L. Xu, W. Zhu, L. Feng, J. Liu, Q. Chen, Z. Dong, J. Zhao, Z. Liu, M. Chen, *Biomaterials* **2018**, *156*, 121.

[2] F. Liu, X. He, H. Chen, J. Zhang, H. Zhang, Z. Wang, *Nat. Commun.* **2015**, *6*, 8003.

[3] L. Z. Feng, L. Cheng, Z. L. Dong, D. L. Tao, T. E. Barnhart, W. B. Cai, M. W. Chen, Z. Liu, *ACS Nano* **2017**, *11*, 927.
